# Reduced risk of cause-specific hospitalisations and all-cause hospitalisation/mortality during treatment with attention-deficit/hyperactivity disorder medications in the course of bipolar disorder: a Swedish registry-based within-subject cohort study

**DOI:** 10.1136/bmjment-2025-302159

**Published:** 2026-03-19

**Authors:** Cagatay Ermis, Antti Tanskanen, Olivier Corbeil, Johannes Lieslehto, Eduard Vieta, Christoph U Correll, Ellenor Mittendorfer-Rutz, Jari Tiihonen, Heidi Taipale

**Affiliations:** 1 Department of Child and Adolescent Psychiatry, Sahlgrenska University Hospital, Gothenburg, Sweden; 2 Department of Forensic Psychiatry, University of Eastern Finland, Niuvanniemi Hospital, Kuopio, Finland; 3 Department of Clinical Neuroscience, Division of Insurance Medicine, Karolinska Institutet, Stockholm, Sweden; 4 Ottawa Hospital Research Institute (OHRI), Clinical Epidemiology Program, SCIENCES Lab, University of Ottawa, Ottawa, Ontario, Canada; 5 Department of Pharmacy, Mental Health University Institute of Quebec, Québec City, Quebec, Canada; 6 Department of Clinical Neuroscience, Karolinska Institutet, Stockholm, Stockholm County, Sweden; 7 Bipolar and Depressive Disorders Unit, Institute of Neuroscience, Hospital Clinic, IDIBAPS, CIBERSAM, University of Barcelona, Barcelona, Spain; 8 Department of Child and Adolescent Psychiatry, Charité - Universitätsmedizin Berlin, Berlin, Germany; 9 Department of Psychiatry and Molecular Medicine, Donald and Barbara Zucker School of Medicine at Hofstra/Northwell, Hempstead, New York, USA

**Keywords:** Bipolar and Related Disorders, Psychopharmacology

## Abstract

**Background:**

Comorbid attention-deficit/hyperactivity disorder (ADHD) increases the burden in bipolar disorder (BD). Concerns about the risk/benefit balance of ADHD treatment have been raised.

**Objective:**

This study aimed to investigate the association between hospital admissions and add-on ADHD medications to antipsychotics and/or mood-stabilisers (APs/MSs) compared with AP/MS alone in BD.

**Methods:**

Individuals with BD prescribed ADHD medications in Sweden during 2006–2021 were identified from national registers of inpatient care, specialised outpatient care, sickness absence and disability pension. ADHD treatment was defined as stimulants (mostly methylphenidate and lisdexamfetamine, rarely amphetamine, dexamphetamine) and non-stimulants (atomoxetine, modafinil). Add-on ADHD treatment to concomitant AP/MS was compared with treatment periods with AP/MS without ADHD treatment, using within-individual models where individuals acted as their own control. Adjusted HRs (aHRs) and CIs (95% CIs) were calculated for the primary outcome of psychiatric hospitalisation, and for the secondary outcomes: substance-use-related, somatic or mania-related hospitalisations, and all-cause hospitalisation/mortality.

**Results:**

Altogether, 17 971 individuals (mean age=32.0±11.6 years, males=37.6%, ADHD=88.9%, follow-up=8.9±4.4 years) with BD who used any ADHD treatment were included. compared with the use of AP/MS alone, add-on stimulant use was related to a lower risk of psychiatric hospitalisations (aHR=0.89, 95% CI 0.85 to 0.93), substance-related hospitalisations (aHR=0.75, 95% CI 0.70 to 0.81) and all-cause hospitalisations/mortality (aHR=0.90, 95% CI 0.87 to 0.93), but was not associated with increased risk for somatic (aHR=1.00, 95% CI 0.90 to 1.12) or mania-related hospitalisations (aHR=0.93, 95% CI 0.72 to 1.20). Of commonly used specific ADHD medications, add-on lisdexamfetamine (aHR=0.81, 95% CI 0.75 to 0.87) and methylphenidate (aHR=0.92, 95% CI 0.88 to 0.97) were associated with decreased risk of psychiatric hospitalisations while add-on atomoxetine was not. Findings on substance-use-related hospitalisations were significant only for stimulants, specifically lisdexamfetamine (aHR=0.70, 95% CI 0.61 to 0.79) and methylphenidate (aHR=0.80, 95% CI 0.74 to 0.86).

**Conclusions:**

Among individuals with BD who received ADHD medications, add-on lisdexamfetamine and methylphenidate were associated with lower risks of psychiatric and substance-use-related hospital admissions, compared with AP/MS use alone. No significant association was found between ADHD medication use and mania-related hospitalisations or somatic admissions when these medications were used together with AP/MS. Larger samples are needed to reach adequate statistical power and conclusive findings on atomoxetine, dexamfetamine and modafinil.

**Clinical implications:**

The findings of this study suggested that the treatment of comorbid ADHD could be considered after adequate mood-stabilisation in patients with BD.

**WHAT IS ALREADY KNOWN ON THIS TOPIC**

- The treatment of attention-deficit/hyperactivity disorder (ADHD) may be associated with improvements in selected clinical outcomes in adequately stabilised patients with bipolar disorder (BD).
- In previous studies, add-on ADHD medications were not related to worsening in mood symptoms after adequate stabilisation with antipsychotics and/or mood-stabilisers (APs/MSs).
- There is limited research regarding comparative effectiveness of adjunctive ADHD medications in the maintenance treatment of BD.

**WHAT THIS STUDY ADDS**

- Compared with treatment with AP/MS alone, add-on ADHD medications were related to fewer psychiatric hospitalisations.
- On the medication level, lisdexamfetamine and methylphenidate were associated with decreased risk of psychiatric hospitalisations; the magnitude was greater with lisdexamfetamine.
- Methylphenidate and lisdexamfetamine were also significantly related to lower risks of substance-use-related hospitalisations.
- There was no significant difference between the two treatment categories concerning somatic hospitalisations and mania-related hospitalisations.

**HOW THIS STUDY MIGHT AFFECT RESEARCH, PRACTICE OR POLICY**

- When used together with AP/MS, lisdexamfetamine and methylphenidate were related to favourable psychiatric real-world outcomes in individuals with BD who received ADHD medications.
- Atomoxetine, dexamfetamine and modafinil should be further evaluated in larger studies.
- Controlled trials are needed to establish their place in clinical treatment and guidelines.

## Background

 Approximately one-sixth of adults with bipolar disorder (BD) also have comorbid attention-deficit/hyperactivity disorder (ADHD).^1^ Comorbid ADHD increases overall illness burden in BD, including more mood episodes, decreased social and occupational functioning, and increased risk for suicide attempts, substance use and anxiety disorders.^2 3^

Add-on methylphenidate, amphetamines, atomoxetine and other non-stimulant treatments can reduce the severity of ADHD-related signs and symptoms in individuals with BD after adequate mood-stabilisation.^4 5^ Several randomised controlled trials (RCTs) also focused on the efficacy of ADHD medications, especially on modafinil, in bipolar depression; however, the use and benefit of modafinil in BD has remained controversial, with conflicting and negative findings from larger RCTs.^6 7^ Moreover, commonly used stimulants (ie, methylphenidate, lisdexamfetamine, amphetamine salts) and atomoxetine were not widely investigated in adults with BD.^4 8 9^ While some previous findings suggested benefits for ADHD signs and symptoms when they were used adjunctively with antipsychotics and/or mood-stabilisers (APs/MSs), high-quality long-term evidence is still lacking in this population.^4 8 9^ However, somatic complications require further attention in the BD population, given the increased risk of mortality due to cardiovascular events and other reasons.^10 11^

Comorbid substance use disorder (SUD) is also related to poorer occupational functioning, a higher number of manic episodes, suicidality and increased mortality risk in BD.^12^,^14^ In addition to studies with BD, previous RCTs on SUD suggested that stimulants can decrease craving in amphetamine-type SUDs and promote sustained abstinence among those with cocaine use.^15 16^ Likewise, in a recent epidemiological study, lisdexamfetamine was related to a lower likelihood of SUD-related hospital admissions and mortality in individuals with amphetamine use disorders.^17^ Given these previous findings on SUD, cotreatment with ADHD medications may further improve the prognosis of BD, specifically reducing illness-related burden and negative outcomes.

Previous literature indicated that stimulants could increase the risk for psychotic symptoms, hypomanic/manic switch and mood destabilisation.^418^,^20^ Similarly, mood destabilisation can also occur during atomoxetine treatment in BD.^4 5 21^ However, a previous registry-based study reported that the risk of manic switch was increased with methylphenidate monotherapy, but not when methylphenidate was added to AP/MS.^19^ Another recent study also extended these findings to depressive episodes and psychiatric hospitalisations, while psychiatric hospitalisations did not differ statistically for methylphenidate use concomitant to AP/MS.^22^ Overall, more observational studies, with large sample sizes and long observation periods, are needed to examine the benefits versus risks of ADHD treatment in BD, as safety concerns are yet to be fully elucidated in this population.^4 8 21^

Despite the potential benefits of ADHD medications for reducing the risk of psychiatric hospitalisation, suicidal behaviour and disability pension/sickness absence in adults with ADHD,^23^ concerns remain about their use in BD regarding the risk of mood destabilisation or emergent psychosis.^4 8^ Accordingly, real-world studies focusing on ADHD medications in people with BD could pave the way for research to better understand the role of ADHD medication in BD, aiming to alleviate illness-related burden.

### Objective

The objective of this study was to investigate the risk of psychiatric hospitalisations related to the add-on use of ADHD medications in BD. Also, secondary aims were to examine substance use, somatic reasons or manic episodes, and all-cause hospitalisation/mortality to evaluate the benefit/risk ratio related to ADHD treatment during the course of BD.

## Methods

### Study sample and design

The study cohort consisted of individuals with BD identified from national Swedish registers: The National Patient Register (ie, data on inpatient and specialised outpatient care) and Microdata for Analyses of Social Insurance (MiDAS register, including disability pensions and sickness absence). Information was also linked from the Prescribed Drug Register (PDR), the Longitudinal Integration Database for Health Insurance and Labour Market Studies and the Cause of Death Register (ie, mortality) (online supplemental table S1). The linkage was conducted via personal identification number, which is assigned to all residents either at birth or on immigration to Sweden.

The inclusion criteria were a diagnosis of BD, using the International Statistical Classification of Diseases and Related Health Problems, 10th Revision (ICD-10) codes of F30–F31, at the age of 16–65 years, residency in Sweden and the presence of ADHD pharmacotherapy during 1 January 2006 to 31 December 2021. The cohort entry was defined as the date of BD diagnosis or 1 January 2006 for individuals with a previous BD diagnosis. January 2006 was chosen because PDR started in July 2005. The base cohort with 105 495 individuals has been described previously.^24^ Of these, 17 971 (17.0%) who used any ADHD treatment at any time interval after a BD diagnosis between 2006 and 2021 were included.

Exclusion criteria were schizophrenia-spectrum disorders (ie, F20–F29) before cohort entry. The follow-up ended at death, migration to another country, a diagnosis of schizophrenia-spectrum disorders (ie, F20–F29) or the end of data linkage (31 December 2021), whichever occurred first.

### Exposures

The PDR (between July 2005 and December 2021) was used to identify dispensed prescription medications during follow-up. Medications were categorised according to the Anatomical Therapeutic Chemical (ATC) classification system (https://atcddd.fhi.no/atc_ddd_index/).^25^ ADHD medications (ATC N06BA) used by the study population were amphetamine, dexamfetamine, methylphenidate, lisdexamfetamine, atomoxetine and modafinil. ADHD medications were further subcategorised into stimulants (N06BA01 amphetamine, N06BA02 dexamfetamine, N06BA04 methylphenidate, N06BA12 lisdexamfetamine) and non-stimulants (ie, N06BA07 modafinil, N06BA09 atomoxetine). APs were defined as N05A (excluding lithium N05AN01), and MSs as valproic acid (N03AG01), lamotrigine (N03AX09), carbamazepine (N03AF01) and lithium (N05AN01). On the other hand, clonidine (C02AC01) and guanfacine (C02AC02) were not studied in the current work because clonidine is not approved for ADHD in Sweden, and guanfacine use is licensed only for children and adolescents, not for adults, leading to an insufficient number of prescriptions in the study population during the follow-up period.

The Swedish PDR records dispensation history from community pharmacies (ie, dates, amount, drug package information [pharmaceutical form, strength per unit, package size, trade name, etc]), which were transformed into medication use periods (ie, when medication use started and ended) to define time-varying exposure categories. In this study, treatment periods were estimated using the validated PRE2DUP (from prescription drug purchases to drug use periods) algorithm,^26^ which converts medication purchase records into drug use periods based on mathematical modelling of personal patterns and guided by expert-defined package-level parameters that ensure clinically meaningful upper and lower limits for daily dose. The PRE2DUP method was implemented to create treatment periods for each specific medication (ATC code) and for each individual by taking into account personal purchase patterns, stockpiling of medications, as well as hospital stays when medication use is not recorded in PDR.^26^ The method is based on the calculation of a sliding temporal average of the daily dose, derived from dispensing dates and amounts. Connecting purchases to form treatment periods is controlled by expert-defined parameters for each drug package that restrict average dose estimates to clinically relevant doses and medication use patterns (ie, termination of treatment and discontinuation of use when the dosage is lower than the minimum threshold). Medication-level treatment periods were post-processed to categorise treatment classes such as stimulants and non-stimulants by combining overlapping use periods of specific medications into these broader categories (for more details, see Exposures in online supplemental material).

ADHD medication use was analysed as adjunctive treatment to AP/MS, based on the treatment recommendations.^5 6^ The reference category in all analyses was the use of AP/MS without concomitant ADHD medications. The main exposures were ADHD medications: stimulants and non-stimulants used concomitantly with AP/MS. The three most common ADHD medications with adequate statistical power, namely methylphenidate, lisdexamfetamine and atomoxetine, were also analysed as separate medication categories. Treatment periods that included add-on stimulants (with or without concomitant non-stimulants) and add-on non-stimulants (excluding concomitant use of stimulants) were separately analysed at a group level.

### Outcomes

#### Primary outcome

The primary outcome of the study was any psychiatric hospitalisation (F00–F99 as the main diagnosis).

#### Secondary outcomes

Secondary outcomes included hospitalisation related to substance use (F10–19, except for F17), somatic hospitalisations (A00–N99, excluding F00–F99) and mania (F30, F31.0, F31.1 and F31.2), as well as all-cause hospitalisation or mortality.

### Statistical analysis

The main analyses included within-individual analyses for the primary outcome and the four secondary outcomes for the five treatment categories in this study. The main analyses were conducted using a within-individual design where each subject acts as his/her own control (for more details, see statistical analysis in online supplemental material). The analyses were conducted with stratified Cox regression models where each person was in a separate stratum to estimate adjusted HR (aHR) and 95% CIs.^17 23^ These models minimise selection bias and automatically eliminate the impact of time-invariant characteristics (eg, sex, genetic characteristics, adverse childhood experiences) by comparing specific treatment periods within the same subjects where time-dependent covariates were adjusted for in the analyses, namely (1) temporal order of treatment categories, (2) time since entry to bipolar cohort, (3) use of antidepressants and (4) benzodiazepines and related drugs (online supplemental table S1).

Several sensitivity analyses were conducted for the primary outcome. First, cotreatment was subdivided by add-on MSs alone, APs alone or APs plus MSs. Second, the analysis was restricted to patients with comorbid ADHD diagnoses. Third, considering that titration and full treatment effect can be achieved only after several weeks, and the effects of exposure may persist after discontinuation of use, another sensitivity analysis was conducted by omitting the first 30 days of all exposure and non-exposure periods. Fourth, the primary outcome was restricted to time periods after the first observed ADHD medication use to ensure that pre-exposure time did not unfairly inflate the results.

A subgroup analysis was conducted for individuals with comorbid SUD (ie, F10–F19, except for F17), as comorbid SUD was related to poorer outcomes in individuals with BD.^12^,^14^ Another subgroup analysis was performed for patients who received a disability pension as a marker of decreased functioning and chronicity. In the subgroup analyses for subjects who had comorbid substance use or disability pension, the follow-up started when the individual had both BD and SUD; or BD and granted disability pension in the corresponding analysis. An individual might receive a related ICD code for SUD or have a disability pension either before the original cohort entry (ie, BD diagnosis) or during the follow-up.

The risk of psychiatric and somatic hospitalisations was analysed in patients ≥30 years of age to facilitate interpretability and comparability of the findings with prior studies.^23 27^ As somatic comorbidities increase by age, these outcomes were also analysed in individuals aged ≥40 years.

Finally, traditional between-individual Cox models were also conducted as a further sensitivity analysis, adjusted for both time-dependent and time-invariant covariates (online supplemental table S1). This analysis compared the risk for the main outcome during add-on ADHD medication use with time periods when AP/MS was used alone (ie, the same reference category). All individuals in the study cohort (n=17 971) contributed to between-individual Cox models, whereas in within-individual models, only individuals who experienced the outcome and had variation in exposure during follow-up contributed directly to the estimates (online supplemental figure S1).

Family-wise error rate (FWER) correction (ie, Bonferroni procedure) was applied to the main analyses in this study. The initial alpha value of 0.05 was adjusted for the five treatment categories and the five study outcomes to 0.05/25 level in the main analyses. After FWER adjustment, uncorrected p values <0.0020 were significant in the main analyses. On the other hand, sensitivity analyses were conducted to explore the robustness of the main findings, and these were not adjusted for multiple comparisons. Only uncorrected p values and 95% CIs were reported. The data analysis was conducted with SAS (V.9.4).

## Results

### Cohort characteristics

The cohort included 17 971 individuals who had a diagnosis of BD and received ADHD medications at any time during follow-up. The mean age of subjects at cohort entry was 32.0±11.6 years (ages 16–17=7.8%), and 37.6% (n=6752) were men (table 1). Additionally, 15 985 (88.9%) received an ADHD diagnosis at some point (online supplemental table S2). The characteristics of the study subgroups are also shown in online supplemental tables S3–S5.

**Table 1:** Sociodemographic and illness characteristics of individuals with BD and ADHD medications at cohort entry

Characteristics	Total sample (n=17 971)
Age at cohort entry, y, M±SD	32.0±11.6
Age at cohort entry, y, median (Q1–Q3)	30 (23–40)
Men, % (n)	37.6 (6752)
Born in Sweden, % (n)	91.5 (16 441)
Sickness absence year before cohort entry, % (n)	31.3 (5619)
Receiving disability pension at cohort entry, % (n)	17.6 (3166)
Previous unipolar depression, % (n)	53.0 (9524)
Previous suicide attempt, % (n)	15.7 (2821)
Time since BD diagnosis at cohort entry <1 year, % (n)	93.0 (16 704)
Anxiety disorders, % (n)	56.6 (10 164)
Substance use disorders, % (n)	28.9 (5197)
Personality disorders, % (n)	17.6 (3160)

ADHD, attention-deficit/hyperactivity disorder; BD, bipolar disorder; M, mean; Q1, first quartile; Q3, third quartile; y, year.

The mean duration of follow-up was 8.9±4.4 years, and 13 624 individuals (75.8%) had at least one treatment interval when they used ADHD medications concomitantly with AP/MS medications. Of them, 90.7% (n=12 361) used stimulant-type add-on ADHD medications with AP/MS, while 23.3% (n=3179) received non-stimulants as cotreatment during the follow-up. The most used add-on ADHD medication was methylphenidate (n=9627, 70.7%), followed by lisdexamfetamine (n=5461, 40.1%) and atomoxetine (n=2470, 18.1%).

### Primary outcome

**Figure 1:**
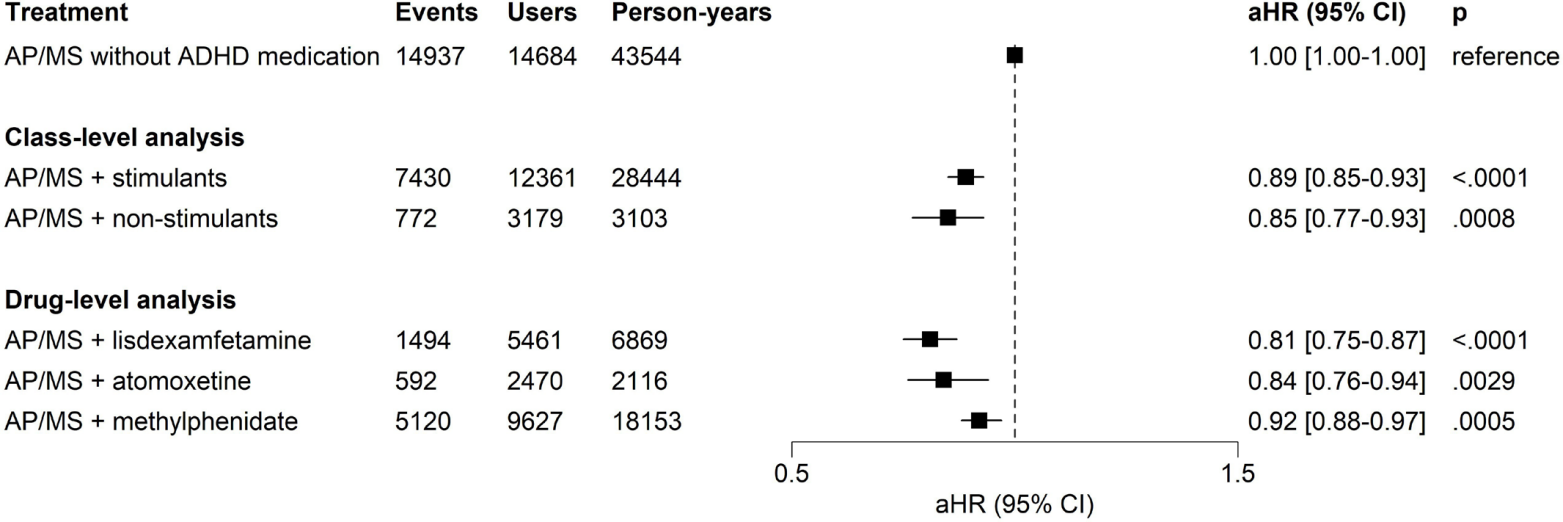
Risk of psychiatric hospital admissions for treatment periods of ADHD medication adjunctive to AP/MS compared to treatment periods with AP/MS alone without any ADHD medication in within-individual analysis. Note: within-individual analysis for treatment periods with both ADHD and BD treatments compared to treatment periods without any ADHD treatment: The number of users was not mutually exclusive for any treatment category since one individual might contribute to various treatment categories in different time frames. The AP/MS category included all combinations or monotherapies of AP and MS medications pooled together. The reference category was treatment periods with BD treatment (ie, AP and/or MS treatment) without ADHD medications. As there were 25 comparisons in the main analyses, the threshold for significance after family-wise error correction was set at p<0.0020 (ie,0.05/25) level. ADHD, attention-deficit/hyperactivity disorder; aHR, adjusted HR; AP, antipsychotic medication; AP/MS, antipsychotic and/or mood-stabiliser medication; BD, bipolar disorder; MS, mood-stabiliser.

In this study, 44.2% (n=7951) of subjects experienced psychiatric hospitalisation at least once. Compared with the use of AP/MS alone, adjunctive stimulant use was associated with a lower risk of psychiatric hospitalisation (aHR=0.89, 95% CI 0.85 to 0.93, p<0.0001) and non-stimulants in general were related to a lower risk of psychiatric hospital admissions (aHR=0.85, 95% CI 0.77 to 0.93, p=0.0008) (figure 1). In medication-level analyses, lisdexamfetamine (aHR=0.81, 95% CI 0.75 to 0.87, p<0.0001) and methylphenidate (aHR=0.92, 95% CI 0.88 to 0.97, p=0.0005) were associated with a decreased risk of psychiatric hospitalisations when combined with AP/MS, while atomoxetine use did not (figure 1).

### Sensitivity analyses for primary outcome

In sensitivity analyses, not adjusted for multiple comparisons, use of stimulants together with MSs alone (aHR=0.84, 95% CI 0.78 to 0.90, p<0.0001) and APs alone (aHR=0.85, 95% CI 0.80 to 0.90, p<0.0001) were associated with decreased psychiatric hospitalisation risk, while this was not the case for combination with both (online supplemental figures S2–S4). Non-stimulants were also associated with a lower risk when added on to MSs alone (aHR=0.76, 95% CI 0.62 to 0.93, p=0.0076), or MSs and APs (aHR=0.81, 95% CI 0.70 to 0.93, p=0.0024), but not when added on to APs alone (aHR=0.97, 95% CI 0.83 to 1.15, p=0.7411).

The primary outcome was also analysed in patients with a comorbid ADHD diagnosis. The main findings remained similar for stimulants (aHR=0.89, 95% CI 0.85 to 0.93, p<0.0001), non-stimulants (aHR=0.83, 95% CI 0.74 to 0.93, p=0.0008), methylphenidate (aHR=0.92, 95% CI 0.88 to 0.97, p=0.0006), lisdexamfetamine (aHR=0.81, 95% CI 0.75 to 0.87, p<0.0001) and atomoxetine (aHR=0.84, 95% CI 0.75 to 0.95, p=0.0038). After omitting the first 30 days of exposure and non-exposure periods in the cohort (n=17 971), stimulants (aHR=0.92, 95% CI 0.88 to 0.97, p=0.0005), non-stimulants (aHR=0.83, 95% CI 0.73 to 0.93, p=0.0013), lisdexamfetamine (aHR=0.86, 95% CI 0.78 to 0.94, p=0.0006) and atomoxetine (aHR=0.85, 95% CI 0.74 to 0.97, p=0.0146) were also associated with a lower risk, while methylphenidate (aHR=0.95, 95% CI 0.90 to 1.00, p=0.0687) was not (online supplemental figure S5). On the other hand, when the analysis was restricted to the periods following the first initiation of ADHD medications (online supplemental figure S6), the findings were significant for stimulants (aHR=0.92, 95% CI 0.88 to 0.97, p=0.0034), methylphenidate (aHR=0.94, 95% CI 0.89 to 1.00, p=0.0431) and lisdexamfetamine (aHR=0.89, 95% CI 0.82 to 0.98, p=0.0115), while not being significant for atomoxetine (aHR=0.88, 95% CI 0.78 to 1.00, p=0.0555) and non-stimulants (aHR=0.90, 95% CI 0.80 to 1.01, p=0.0756).

Sensitivity analyses for the primary outcome among individuals with comorbid SUD were consistent with the main findings (online supplemental figure S7). Additionally, findings on stimulants and lisdexamfetamine remained significant in the patient subgroup who received disability pension (online supplemental figure S8) and among individuals aged ≥30 years at cohort entry (online supplemental figure S9). The findings were in line with the main results when the analysis was restricted to individuals aged between 16 and 29 years (online supplemental figure S10). In addition, stimulants (aHR=0.83, 95% CI 0.76 to 0.91, p<0.0001), lisdexamfetamine (aHR=0.66, 95% CI 0.53 to 0.81, p<0.0001) and methylphenidate (aHR=0.88, 95% CI 0.80 to 0.97, p=0.0111) were associated with lower risk of psychiatric hospitalisations in the 4644 individuals aged ≥40 years at cohort entry, while non-stimulants (aHR=0.93, 95% CI 0.76 to 1.13, p=0.4635) and atomoxetine (aHR=1.00, 95% CI 0.76 to 1.31, p=0.9766) were not.

Finally, in traditional between-individual Cox regression models, implemented as sensitivity analyses, the findings for lisdexamfetamine (aHR=0.76, 95% CI 0.71 to 0.82, p<0.0001), methylphenidate (aHR=0.91, 95% CI 0.87 to 0.96, p=0.0002) and stimulants at a class-level (aHR=0.86, 95% CI 0.83 to 0.90, p<0.0001) were related to a lower risk, while this was not the case with atomoxetine (aHR=0.92, 95% CI 0.80 to 1.05, p=0.2279) and non-stimulants (aHR=0.89, 95% CI 0.80 to 1.00, p=0.0512).

### Secondary outcomes

**Figure 2:**
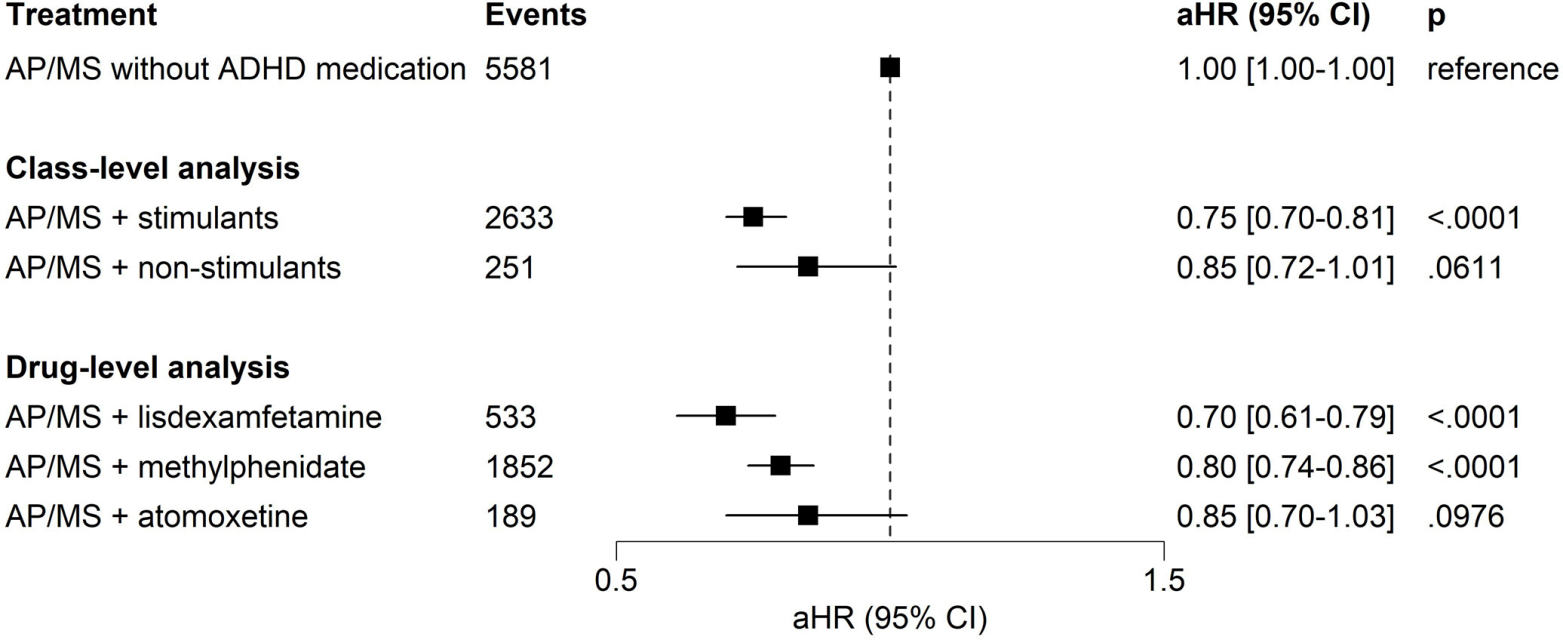
Risk of substance-use-related hospital admissions for treatment periods of ADHD medication adjunctive to AP/MS compared to treatment periods with AP/MS alone without any ADHD medication in within-individual analysis. Note: within-individual analysis for treatment periods with both ADHD and BD treatments compared to treatment periods without any ADHD treatment: The number of users was not mutually exclusive for any treatment category since one individual might contribute to various treatment categories in different time frames. The AP/MS category included all combinations or monotherapies of AP and MS medications pooled together. The reference category was treatment periods with BD treatment (ie, AP and/or MS treatment) without ADHD medications. As there were 25 comparisons in the main analyses, the threshold for significance after family-wise error rate correction was set at p<0.0020 (ie, 0.05/25) level. ADHD, attention-deficit/hyperactivity disorder; aHR, adjusted HR; AP, antipsychotic medication; AP/MS, antipsychotic and/or mood stabiliser medication; BD, bipolar disorder; MS, mood-stabiliser.

During follow-up, 21.5% (n=3860) had a substance-use-related hospitalisation. A lower risk of substance-use-related hospitalisation was observed for the treatment periods with add-on stimulant medications (aHR=0.75, 95% CI 0.70 to 0.81, p<0.0001) compared with non-use of ADHD medications (figure 2). At the drug-level, the findings were similar for the treatment periods with lisdexamfetamine (aHR=0.70, 95% CI 0.61 to 0.79, p<0.0001) and methylphenidate (aHR=0.80, 95% CI 0.74 to 0.86, p<0.0001), but not with atomoxetine (aHR=0.85, 95% CI 0.70 to 1.03, p=0.0976) or non-stimulants in general (aHR=0.85, 95% CI 0.72 to 1.01, p=0.0611).

**Figure 3:**
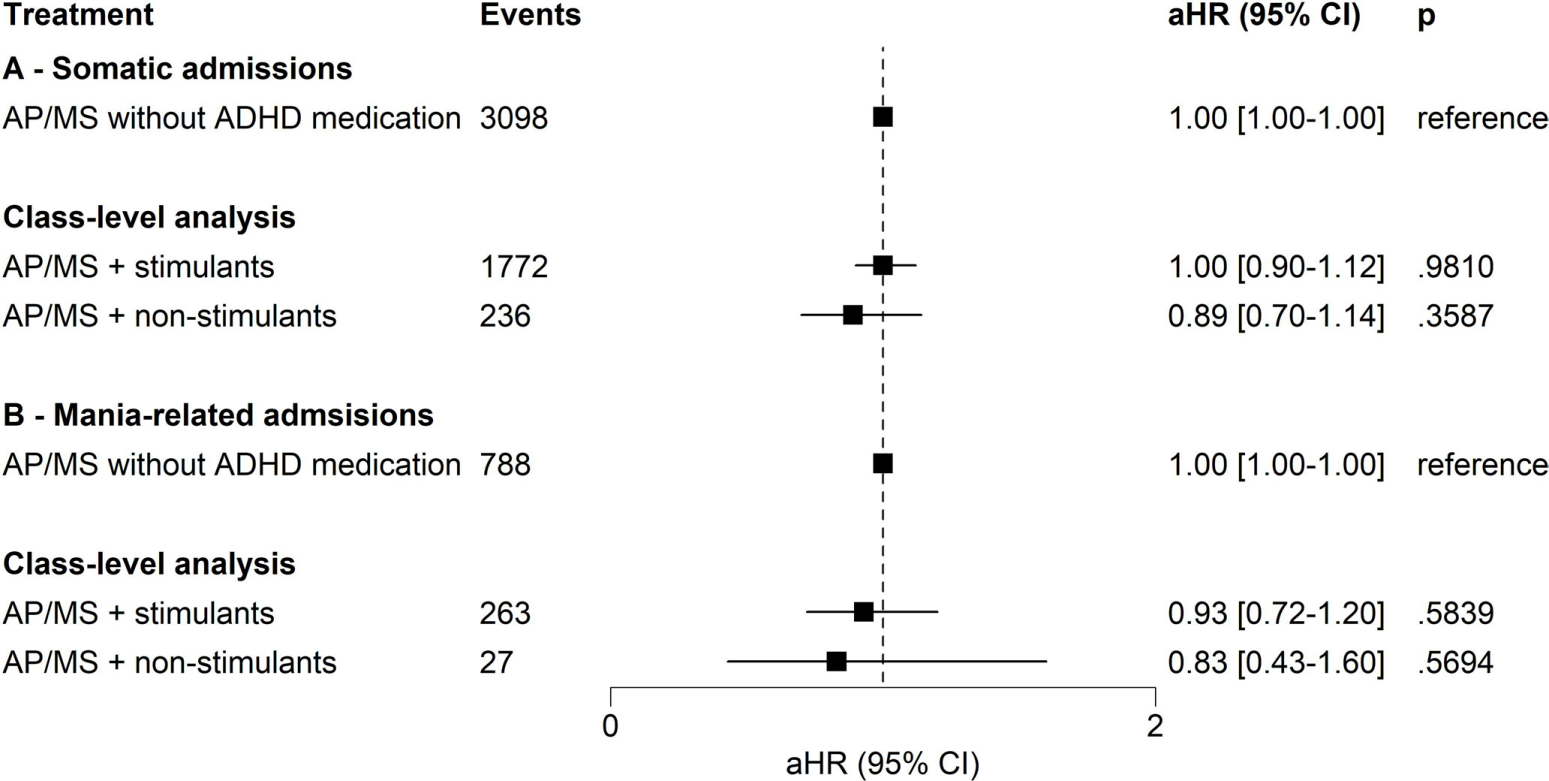
Risk of somatic hospital admissions (**A**) and mania-related hospital admissions (**B**) for treatment periods of ADHD medication adjunctive to AP/MS compared to treatment periods with AP/MS alone without any ADHD medication in within-individual analysis. Note: within-individual analysis for treatment periods with both ADHD and BD treatments compared to treatment periods without any ADHD treatment: The number of users was not mutually exclusive for any treatment category since one individual might contribute to various treatment categories in different timeframes. The AP/MS category included all combinations or monotherapies of AP and MS medications pooled together. The reference category was treatment periods with BD treatment (ie, AP and/or MS treatment) without ADHD medications. As there were 25 comparisons in the main analyses, the threshold for significance after family-wise error rate correction was set at p<0.0020 (ie, 0.05/25) level. ADHD, attention deficit/hyperactivity disorder; aHR, adjusted HR; AP, antipsychotic medication; AP/MS, antipsychotic and/or mood-stabiliser medication; BD, bipolar disorder; MS, mood-stabiliser.

Approximately one-fourth of the cohort (n=4646, 25.9%) had somatic hospitalisations during follow-up. No treatment category in the study was related to somatic hospitalisations in class-level analyses (figure 3), and in medication-level analyses (ie, methylphenidate, aHR=1.04, 95% CI 0.92 to 1.17, p=0.5417; lisdexamfetamine, aHR=0.85, 95% CI 0.69 to 1.06, p=0.1516; and atomoxetine, aHR=0.79, 95% CI 0.56 to 1.11, p=0.1758). The findings on somatic hospitalisations were similar when the analyses were stratified to those aged 16–29 and ≥30 years at cohort entry (online supplemental figures S11 and S12). Also, the findings on stimulants (aHR=1.07, 95% CI 0.89 to 1.29, p=0.4901), non-stimulants (aHR=1.03, 95% CI 0.74 to 1.44, p=0.8609), methylphenidate (aHR=1.08, 95% CI 0.88 to 1.32, p=0.4831), lisdexamfetamine (aHR=1.21, 95% CI to 0.84 to 1.72, p=0.3074) and atomoxetine (aHR=0.81, 95% CI 0.47 to 1.39, p=0.4409) remained in line with the main findings when the analysis was restricted to the 4644 patients with ≥40 years of age at cohort entry.

Altogether, 939 individuals (5.2%) experienced mania-related hospitalisation during the follow-up. Add-on treatment with stimulants (aHR=0.93, 95% CI 0.72 to 1.20, p=0.5839) or non-stimulant medications (aHR=0.83, 95% CI 0.43 to 1.60, p=0.5694) was not associated with mania-related hospital admissions (figure 3), nor was add-on treatment with methylphenidate (aHR=0.95, 95% CI 0.71 to 1.27, p=0.7364), lisdexamfetamine (aHR=0.93, 95% CI 0.56 to 1.54, p=0.7776) or atomoxetine (aHR=1.34, 95% CI 0.59 to 3.07, p=0.4867).

Finally, all-cause hospitalisation or mortality was also another secondary outcome that occurred in 12 243 (68.1%) individuals. The findings for stimulants (aHR=0.90, 95% CI 0.87 to 0.93, p<0.0001), lisdexamfetamine (aHR=0.86, 95% CI 0.80 to 0.92, p<0.0001) and methylphenidate (aHR=0.93, 95% CI 0.89 to 0.96, p=0.0001) were statistically significant (online supplemental figure S13).

## Discussion

The present nationwide, within-subject cohort study aimed to fill the gap in the literature concerning the use of ADHD medications in BD adjunctive to MSs (ie, lithium or antiseizure medications) and APs. Specifically, concomitant use of stimulant medications was related to decreased risks of psychiatric and substance-use-related hospitalisations, compared with periods with BD medications alone in the same individual. Importantly, stimulant medications were associated with a reduced risk of substance-use-related hospitalisations, while this finding was not statistically significant for the treatment periods with non-stimulants or atomoxetine. Of the three medications studied at the drug level, lisdexamfetamine had the most robust findings for a reduced risk of both any psychiatric and substance-use-related hospitalisations. Finally, no ADHD medication was associated with mania-related and somatic hospitalisations and treatment with commonly used stimulant medications added on to AP/MS was also related to a decreased risk of all-cause hospitalisation or mortality.

Notably, the current findings reflect only the population of patients receiving adjunctive ADHD medications to concomitant AP/MS treatment. Thus, these results may apply only to a relatively stable subgroup of individuals and may therefore not be generalised to individuals with unstable or untreated BD.

In line with previous registry-based studies on add-on methylphenidate with AP/MS treatment,^19 22^ the main findings of this study suggested stimulant use was associated with a lower risk of psychiatric hospitalisations without any risk increase in mania-related admissions. The current study also focused on other commonly used ADHD medications, namely lisdexamfetamine and atomoxetine. Specifically, lisdexamfetamine was associated with decreased risks of the studied outcomes, in line with previous registry-based studies with ADHD or amphetamine use disorder.^17 23^ Nevertheless, the sample size did not provide high statistical power for atomoxetine, given the adjustment for multiple testing. Accordingly, atomoxetine, modafinil, dexamfetamine and amphetamine salts should be further investigated in larger samples to reach conclusive results.

Consistent with previous observational studies and RCTs,^9 19 22 28^ the findings of this study did not suggest any significant association between ADHD medications and manic switch, somatic complications or clinical deterioration when patients are treated with concomitant BD medications. However, it is important to emphasise that these outcomes represent only severe events that require hospitalisation, excluding milder somatic adverse events and hypomania during outpatient follow-up.

In this cohort of 105 495 individuals with BD,^24^ 17.0% received ADHD medications at some time point during follow-up, and the current findings were obtained from these individuals. Thus, one should interpret our findings with caution, as it is possible that the current findings reflect relatively stable patients and may not necessarily involve the most severe or unstable forms of BD, as physicians in real-world practice might avoid prescribing ADHD medications when they consider increased risk for mood-destabilisation or manic switch.^4 8^

ADHD medications could lead to a long-term increase in hypertension and cardiovascular risk,^29^ requiring further attention in the BD population, who have an increased risk of mortality due to cardiovascular/cerebrovascular events, respiratory diseases or infections and other causes,^10 11^ and given an even higher risk of all-cause mortality with the presence of comorbid substance use.^14^ The findings of the present study did not reveal any relationship between somatic hospitalisations and the use of ADHD medications; on the contrary, all-cause hospitalisation or mortality was less common during treatment with ADHD medications. Despite the relatively long follow-up, the current findings did not consider potential cumulative effects of medications over the long term for somatic complications, as the current study focused on somatic hospitalisations as repeated events during the treatment, not as a one-time event. Also, it should be noted that the study population was relatively young, with a median age of 30 years at cohort entry and that possible risks of somatic events may increase with age. Nevertheless, the sensitivity analysis conducted based on age groups did not show any increase in somatic admissions among older individuals, with a maximum age of 65 years. Larger samples and longer follow-up periods should re-examine these findings.

The decreased risk of substance-use-related hospitalisations with ADHD medications is also consistent with previous observational and interventional studies in patients with ADHD or SUD,^15^,^1730^ while this finding was not observed with non-stimulants. In addition, 28.9% of the current cohort had a SUD at entry. Accordingly, the finding on substance-related hospitalisations is a key study outcome, as comorbid SUD poses a major burden to patients, potentially complicating the illness course.^12^,^14^ Thus, the current findings suggesting reductions in substance-use-related hospitalisations have important clinical implications, also countering previous concerns that use of stimulants may increase substance-use-related adverse outcomes and may be contraindicated in the presence of comorbid SUD in people with BD.^4^

The study has some limitations that should be considered when interpreting the results. First, despite using the within-individual design, time-related confounding beyond adjusted covariates (ie, time-varying use of other psychotropic drugs, temporal order of treatments and time since cohort entry) was a limitation of the current study. Furthermore, information on important clinical factors such as baseline severity and fluctuation of mood symptoms could not be taken into account, and it should be noted that it is common practice to prescribe ADHD treatments mainly during the stable maintenance phase of BD, when clinical stability in mood symptoms or substance use is achieved.^4 6 8^ Therefore, clinical stability often precedes treatment-onset or reinitiation of ADHD medications in real-world practice, and the time-varying impact of clinical stability could pose residual confounding in the results, as discussed in detail previously.^22^ However, in view of the follow-up time of up to fifteen years, it is relatively unlikely that ADHD medication use and possible substance use could be monitored in routine clinical practice so closely that treatment periods could only represent a clinically stable phase. Second, one-sixth of patients with BD received ADHD medications during the follow-up, and the severity of mood symptoms was not available. However, results were confirmed in the subgroup of patients on disability pension, a marker of illness chronicity and severity. Third, modafinil, atomoxetine, dexamfetamine and amphetamine salts were not sufficiently widely used in Sweden to reach conclusive results at an individual drug level for these medications. Accordingly, results on stimulants mainly represent the use of methylphenidate and lisdexamfetamine. Additionally, alpha-2 adrenergic agonists (ie, clonidine and guanfacine) were not included in this current study. Fourth, treatment adherence can only be estimated based on personal dispensing patterns from administrative registers, which do not directly include information on treatment adherence. Abrupt discontinuation of ADHD medications can lead to a misclassification problem (ie, when worsening and related hospitalisation after treatment cessation was counted as occurring during the prior treatment period); thus, the current estimates obtained from PRE2DUP were conservative to avoid overestimation of effectiveness.

Finally, although individuals with BD at ages 16 and 17 were included in the study cohort, this subgroup was too small to assess the impact of add-on ADHD medications to AP/MS in the paediatric subgroup. Since ADHD is even more common in youth than adults, future studies should also focus on ADHD medication use in children and adolescents with BD.

In conclusion, the current findings suggest that the use of stimulants as an adjunctive treatment to AP/MS in individuals with BD is associated with several positive outcomes, including decreased risk of psychiatric hospitalisation and substance-use-related hospitalisations without any risk increase in mania-related or somatic hospitalisations. Larger samples and high-quality RCTs are needed to reach more generalisable conclusions for treatment guidelines.

## Supplementary material

10.1136/bmjment-2025-302159 online supplemental file 1

## Data Availability

No data are available.
