# Supplementary material for: Reduced risk of cause-specific hospitalisations and all-cause hospitalisation/mortality during treatment with attention-deficit/hyperactivity disorder medications in the course of bipolar disorder: a Swedish registry-based within-subject cohort study
Source: BMJ Ment Health. 2026 Mar 19;29(1):e302159. doi: 10.1136/bmjment-2025-302159 (PMC13034203; doi:10.1136/bmjment-2025-302159)
Supplement: online supplemental file 1 [file bmjment-29-1-s001.docx]

**Supplemental Material**

**Table of Contents**

**Supplemental Methods**

**References**

**Table S1.** Covariate definitions in within-individual and between-individuals models implemented in the study

**Table S2.** Sociodemographic and illness characteristics of individuals with bipolar disorder, comorbid ADHD diagnosis and treatment

**Table S3.** Sociodemographic and illness characteristics of individuals with bipolar disorder, ADHD treatment, and substance use disorder

**Table S4.** Sociodemographic and illness characteristics of individuals with bipolar disorder, ADHD treatment, and disability pension

**Table S5.** Sociodemographic and illness characteristics of individuals aged ≥30 years

**Figure S1.** Illustration of the encoding of variables, exposures and outcomes and within-individual design

**Figure S2.** Sensitivity analysis for risk of psychiatric hospital admissions associated with use of attention-deficit/hyperactivity disorder (ADHD) medications in addition to mood-stabilizers in within-individual analysis. The reference category was use of bipolar disorder treatment (i.e., antipsychotics or mood stabilizers [AP/MS]) without concomitant ADHD medications

**Figure S3.** Sensitivity analysis for risk of psychiatric hospital admissions associated with use of attention-deficit hyperactivity disorder (ADHD) medications in addition to antipsychotic medications in within-individual analysis. The reference category was use of bipolar disorder treatment (i.e., antipsychotics or mood stabilizers [AP/MS]) without concomitant ADHD medications

**Figure S4.** Sensitivity analysis for risk of psychiatric hospital admissions associated with use of attention-deficit hyperactivity disorder (ADHD) medications in addition to mood-stabilizers and antipsychotics in within-individual analysis. The reference category was use of bipolar disorder treatment (i.e., antipsychotics or mood stabilizers [AP/MS]) without concomitant ADHD medications

**Figure S5.** Sensitivity analysis for risk of psychiatric hospital admissions for treatment periods of ADHD medication in addition to antipsychotics or mood stabilizers (AP/MS) compared to treatment periods with AP/MS alone without any ADHD medication in within-individual analysis after omitting the first 30 days of exposure and non-exposure periods in the whole cohort

**Figure S6.** Sensitivity analysis for risk of psychiatric hospital admissions for treatment periods of ADHD medication in addition to antipsychotics or mood stabilizers (AP/MS) compared to treatment periods with AP/MS alone without any ADHD medication in within-individual analysis by restricting the analysis to time periods after the first observed ADHD medication use

**Figure S7**. Sensitivity analysis for risk of psychiatric hospitalizations associated with use of add-on stimulant and non-stimulant medications compared to treatment periods with antipsychotic and/or mood-stabilizer alone (AP/MS) on group-level analysis in within-individual models including individuals who had comorbid substance use disorder during the cohort

**Figure S8**. Sensitivity analysis for risk of psychiatric hospitalizations associated with use of add-on stimulant and non-stimulant medications compared to treatment periods with antipsychotic and/or mood-stabilizer alone (AP/MS) on group-level analysis in within-individual models including individuals who received disability pension during the cohort

**Figure S9.** Risk of psychiatric hospital admissions for treatment periods of ADHD medication in addition to antipsychotics or mood stabilizers (AP/MS) compared to treatment periods with AP/MS alone without any ADHD medication in within-individual analysis including individuals aged ≥30 years at the cohort entry

**Figure S10.** Risk of psychiatric hospital admissions for treatment periods of ADHD medication in addition to antipsychotics or mood stabilizers (AP/MS) compared to treatment periods with AP/MS alone without any ADHD medication in within-individual analysis including individuals aged 16-29 years at the cohort entry

**Figure S11.** Risk of somatic hospital admissions for treatment periods of ADHD medication in addition to antipsychotics or mood stabilizers (AP/MS) compared to treatment periods with AP/MS alone without any ADHD medication in within-individual analysis including individuals aged ≥30 years at the cohort entry

**Figure S12.** Risk of somatic hospital admissions for treatment periods of ADHD medication in addition to antipsychotics or mood stabilizers (AP/MS) compared to treatment periods with AP/MS alone without any ADHD medication in within-individual analysis including individuals aged 16-29 years at the cohort entry

**Figure S13.** Risk of all-cause hospitalization/ mortality for treatment periods of ADHD medication adjunctive to antipsychotics or mood stabilizers (AP/MS) compared to treatment periods with AP/MS alone without any ADHD medication in within-individual analysis.

**Supplemental Methods**

**Exposure**

The PRE2DUP method was implemented to create treatment periods for each specific study medication, defined by Anatomical Therapeutic Chemical (ATC) code, and separately for each individual in the study population. The post-processing feature of PRE2DUP was utilized to group medications into treatment categories used in the study. Post-processing combined overlapping use of these categories to define time periods of ‘add-on’ treatment (i.e. attention-deficit hyperactivity disorder [ADHD] medications used together with antipsychotics and/or mood stabilizers [AP/MS]) and the reference category of AP/MS alone, without concomitant ADHD drugs.

Previous studies have shown that validity of PRE2DUP method is good or very good for medications that are prescribed as standing treatment, as opposed to ‘as needed’ medications. PRE2DUP has been validated using different methodologies, based on interview ^1^, expert-opinion ^2^, and forensic toxicology ^3^.

**Statistical Analysis**

The main aim of this study design was to compare treatments periods within the same individuals. The comparisons in the current study were conducted for ADHD medications when they were added to ongoing AP/MS medications. The reference category in all analyses was the use of BD treatment without any concomitant ADHD medications (i.e., AP/MS without ADHD medications).

Stratified Cox regression analyses using a within-individual model was the main analysis where treatment periods were compared (i.e., on-medication vs. off-medication periods for a particular treatment) within the same individual. That is, one forms his or her own stratum, and time is reset after the outcome event in the stratum. This model minimizes potential selection bias (i.e. patients who receive a certain medication might differ from those receiving another treatment) by comparing treatment periods within the same individual. An illustration of the dataset was shown in Figure S1 and the details of within-individual models were described in detail elsewhere ^4^.

On the other hand, in between-individual models, repeated outcome was handled by robust variance estimator for calculation of confidence intervals.

**References**

1. Taipale H, Tanskanen A, Koponen M, et al. Agreement between PRE2DUP register data modeling method and comprehensive drug use interview among older persons. *Clin Epidemiol* 2016;8:363-71. doi: 10.2147/CLEP.S116160

2. Tanskanen A, Taipale H, Koponen M, et al. Drug exposure in register-based research-An expert-opinion based evaluation of methods. *PLoS One* 2017;12(9):e0184070. doi: 10.1371/journal.pone.0184070

3. Forsman J, Taipale H, Masterman T, et al. Comparison of dispensed medications and forensic-toxicological findings to assess pharmacotherapy in the Swedish population 2006 to 2013. *Pharmacoepidemiol Drug Saf* 2018;27(10):1112-22. doi: 10.1002/pds.4426

4. Allison PD. Fixed effects regression models: SAGE Publications, Inc. 2009.

**Table S1.** Covariate definitions in within-individual and between-individuals models implemented in the study

| Covariate | Definition | Register source | Models |
| --- | --- | --- | --- |
| Order of treatments | Temporal order of treatments, continuously updated in the models and categorized as first, second, third and >third | PDR | W, B |
| Time since cohort entry | Continuously updated in the models, categorized as 0-1, 1-3 and >3 years |  | W |
| Antidepressants | Continuously updated in the models, ATC N06A | PDR | W, B |
| Benzodiazepines and related drugs | Continuously updated in the models, ATC N05BA, N05CD, N05CF | PDR | W, B |
| Age | Age at cohort entry | LISA | B |
| Gender | Man vs. woman | LISA | B |
| Disability pension | Continuously updated in the models, no vs. yes after the first granted decision. | MiDAS | B |
| Number of previous hospitalizations due to bipolar disorder | At cohort entry, categorized as 0, 1, 2-3 vs. >3 | NPR | B |
| Personality disorders | Continuously updated in the models with status ”no” until the first diagnosis ICD-10 F60-F69 occurred and ”yes” thereafter. | NPR | B |
| Cardiovascular disease | Continuously updated in the models with status ”no” until the first diagnosis ICD-10 I00-I99 occurred and ”yes” thereafter. | NPR | B |
| Diabetes | Continuously updated in the models with status ”no” until the first diagnosis ICD-10 E10-E14 occurred and ”yes” thereafter. | NPR | B |
| Previous suicide attempt | Continuously updated in the models with status ”no” until the first diagnosis ICD-10 X60-X84, Y10-Y34 occurred and ”yes” thereafter. | NPR | B |
| Previous accidental overdose | Continuously updated in the models with status ”no” until the first diagnosis ICD-10 X40-X49 occurred and ”yes” thereafter. | NPR | B |
| *Abbreviations:* ATC=Anatomical Therapeutic Chemical classification, B=between-individual models, ICD-10=International Classification of Diseases - Tenth Revision, LISA=The Longitudinal Integration Database for Health Insurance and Labor Market Studies, MiDAS=Microdata for Analyses of Social Insurance, NPR=National Patient register, PDR=Prescribed Drug Register, W= within-individual models. | | | |

**Table S2.** Sociodemographic and illness characteristics of individuals with bipolar disorder, comorbid ADHD diagnosis and treatment

| Characteristics ^a^ | Total sample ^b^  (n=15,985) |
| --- | --- |
| Age at cohort entry, y, M ± SD  Age at cohort entry, y, median (Q1-Q3) | 31.2 ± 11.2  29 (22 – 39) |
| Men, % (n) | 37.5 (5,992) |
| Born in Sweden, % (n) | 91.6 (14,638) |
| Sickness absence year before cohort entry, % (n) | 30.4 (4,852) |
| Receiving disability pension at cohort entry, % (n) | 16.9 (2,708) |
| Previous unipolar depression, % (n) | 53.0 (8,476) |
| Previous suicide attempt, % (n) | 15.9 (2,548) |
| BD diagnosis < 1 year at cohort entry, % (n) ^a^ | 94.2 (15,051) |
| Anxiety disorders, % (n)  Substance use disorders, % (n) | 58.2 (9,309)  30.1 (4,808) |
| Personality disorders, % (n) | 18.1 (2,895) |

*Note*. ADHD=attention-deficit/hyperactivity disorder, BD=bipolar disorder, IQR= interquartile range, M= mean, Q1=first quartile, Q3=third quartile, SD= standard deviation, y= year.

^a^ Based on the original cohort entry: At time of BD diagnosis or 1st January 2006 for those who had diagnosis before 2006

^b^ The subgroup was formed by individuals who received comorbid ADHD diagnosis at some point. One might receive comorbid ADHD diagnosis either before the original cohort entry (i.e. bipolar disorder diagnosis) or during the follow-up.

**Table S3.** Sociodemographic and illness characteristics of individuals with bipolar disorder, ADHD treatment, and substance use disorder

| Characteristics ^a^ | Total sample ^b^  (n=7,765) |
| --- | --- |
| Age at cohort entry, y, M ± SD  Age at cohort entry, y, median (Q1-Q3) | 32.0 ± 11.2  30 (23-40) |
| Men, % (n) | 43.5 (3,374) |
| Born in Sweden, % (n) | 92.0 (7,141) |
| Sickness absence year before cohort entry, % (n) | 31.1 (2,411) |
| Receiving disability pension at cohort entry, % (n) | 20.7 (1,607) |
| Previous unipolar depression, % (n) | 57.8 (4,484) |
| Previous suicide attempt, % (n) | 27.3 (2,119) |
| BD diagnosis < 1 year at cohort entry, % (n) ^a^ | 91.9 (7,139) |
| Anxiety disorders, % (n) | 64.5 (5,008) |
| Personality disorders, % (n) | 24.9 (1,934) |

*Note*. ADHD=attention-deficit/hyperactivity disorder, BD=bipolar disorder, IQR= interquartile range, M= mean, Q1=first quartile, Q3=third quartile, SD= standard deviation, y= year.

^a^ Based on the original cohort entry: At time of BD diagnosis or 1st January 2006 for those who had diagnosis before 2006

^b^ The subgroup was formed by individuals who had SUD. One might receive related ICD-code either before the original cohort entry (i.e., bipolar disorder diagnosis) or during the follow-up. Therefore, the number of individuals is higher than the number of those at the original cohort entry. In this subgroup analysis, the follow-up started when an individual had both bipolar disorder and substance use disorder diagnoses: The 1st of January 2006 for those who had both bipolar disorder and substance use disorder diagnoses before 2006, or the specific time point when both conditions were present.

**Table S4.** Sociodemographic and illness characteristics of individuals with bipolar disorder, ADHD treatment, and disability pension

| Characteristics ^a^ | Total sample ^b^  (n=7,356) |
| --- | --- |
| Age at cohort entry, y, M ± SD  Age at cohort entry, y, median (Q1-Q3) | 33.3 ± 12.9  31 (22-43) |
| Men, % (n) | 33.6 (2,468) |
| Born in Sweden, % (n) | 92.3 (6,786) |
| Previous unipolar depression, % (n) | 55.2 (4,057) |
| Previous suicide attempt, % (n) | 20.3 (1,491) |
| BD diagnosis < 1 year at cohort entry, % (n) ^a^ | 86.6 (6,373) |
| Anxiety disorders, % (n) | 58.4 (4,299) |
| Substance use disorders, (n) | 31.0 (2,283) |
| Personality disorders, % (n) | 24.3 (1,787) |

*Note*. ADHD=attention-deficit/hyperactivity disorder, BD=bipolar disorder, IQR= interquartile range, M= mean, Q1=first quartile, Q3=third quartile, SD= standard deviation, y= year.

^a^ Based on the original cohort entry: At time of BD diagnosis or 1st January 2006 for those who had diagnosis before 2006

^b^ The subgroup was formed by individuals who received disability pension. One might receive disability pension either before the original cohort entry (i.e. bipolar disorder diagnosis) or during the follow-up. Therefore, the number of individuals is higher than the number of those at the original cohort entry. In this subgroup analysis, the follow-up started when one individual had both bipolar disorder diagnosis and disability pension: The 1st of January 2006 for those who had both bipolar disorder and disability pension before 2006, or the specific time point when both conditions were met.

**Table S5.** Sociodemographic and illness characteristics of individuals aged ≥30 years

| Characteristics ^a^ | Total sample  (n=9,389) |
| --- | --- |
| Age at cohort entry, y, M ± SD  Age at cohort entry, y, median (Q1-Q3) ^a^ | 40.9 ± 8.4  39 (34 – 46) |
| Men, % (n) | 41.6 (3,901) |
| Born in Sweden, % (n) | 89.2 (8,379) |
| Sickness absence year before cohort entry, % (n) | 41.1 (3,862) |
| Receiving disability pension at cohort entry, % (n) | 21.4 (2,013) |
| Previous unipolar depression, % (n) | 54.4 (5,105) |
| Previous suicide attempt, % (n) | 14.6 (1,371) |
| BD diagnosis < 1 year at cohort entry, % (n) | 89.5 (8,404) |
| Anxiety disorders, % (n) | 55.2 (5,183) |
| Substance use disorders, (n) | 30.6 (2,868) |
| Personality disorders, % (n) | 18.1 (1,696) |

*Note*. ADHD=attention-deficit/hyperactivity disorder, BD=bipolar disorder, IQR= interquartile range, M= mean, Q1=first quartile, Q3=third quartile, SD= standard deviation, y= year.

^a^ Based on the original cohort entry: At time of BD diagnosis or 1st January 2006 for those who had diagnosis before 2006


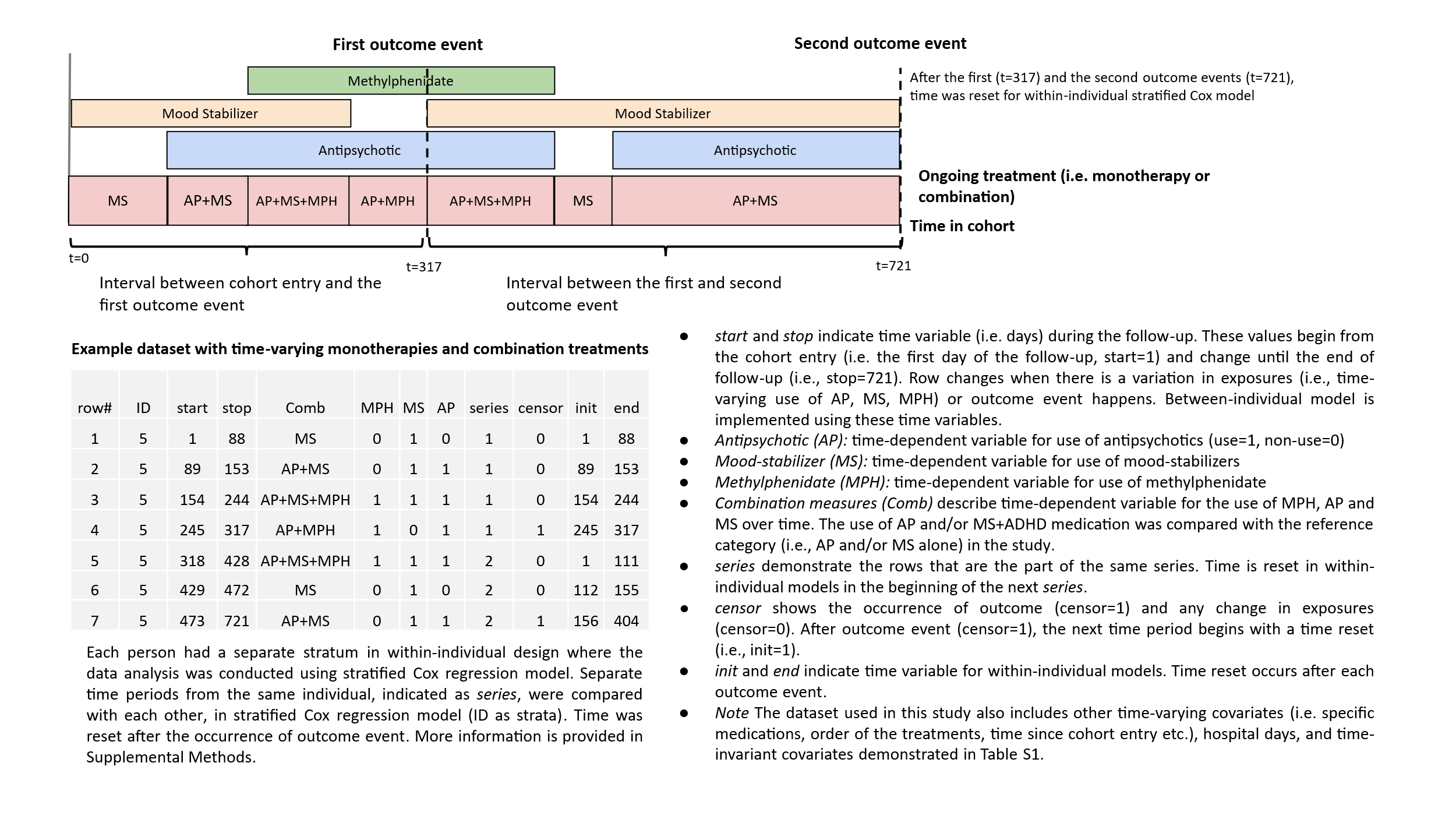


**Figure S1.** Illustration of the encoding of variables, exposures and outcomes and within-individual design

Abbreviations: ADHD=attention-deficit/hyperactivity disorder, AP=antipsychotics, MPH=methylphenidate, MS=mood-stabilizers

**
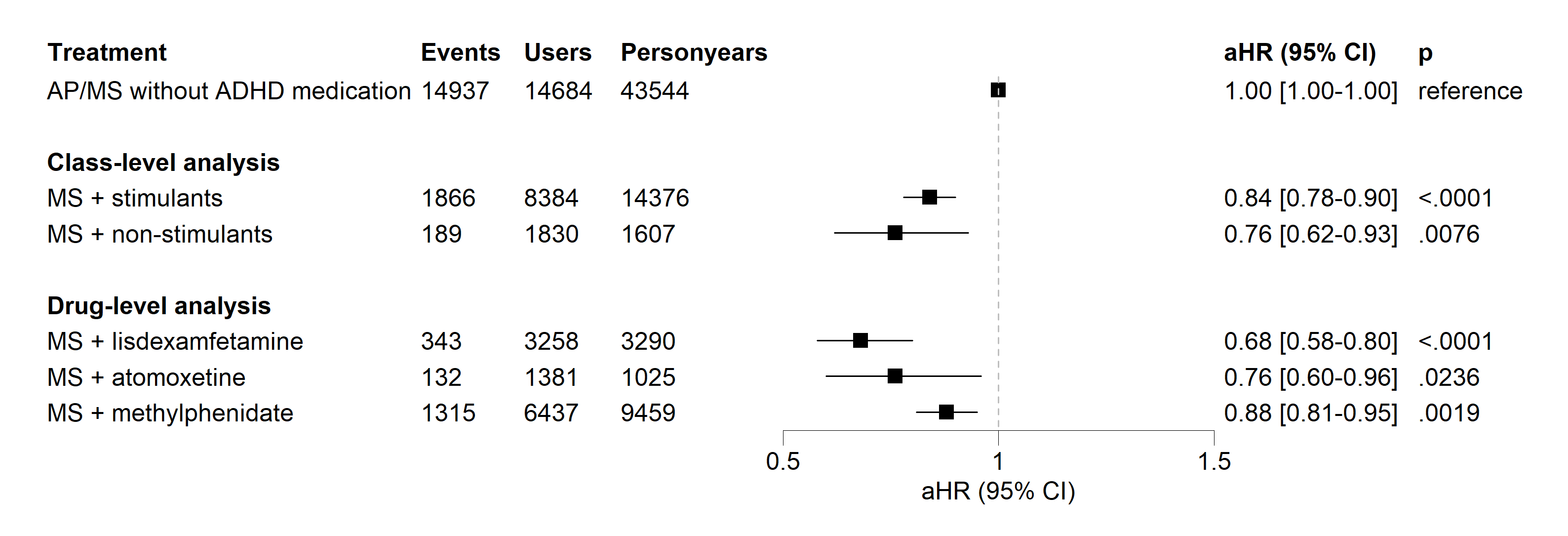
**

**Figure S2.** Sensitivity analysis for risk of psychiatric hospitalizations associated with use of attention-deficit/hyperactivity disorder (ADHD) treatment in addition to treatment with mood-stabilizers in within-individual analysis. The reference category was use of bipolar disorder treatment (i.e., antipsychotics or mood stabilizers [AP/MS]) without concomitant ADHD medications

*Abbreviations:* ADHD=attention-deficit/hyperactivity disorder, aHR=adjusted hazard ratio, AP=antipsychotic medications, AP/MS=antipsychotic and/or mood-stabilizer medication, CI=confidence interval, MS=mood-stabilizers.

**
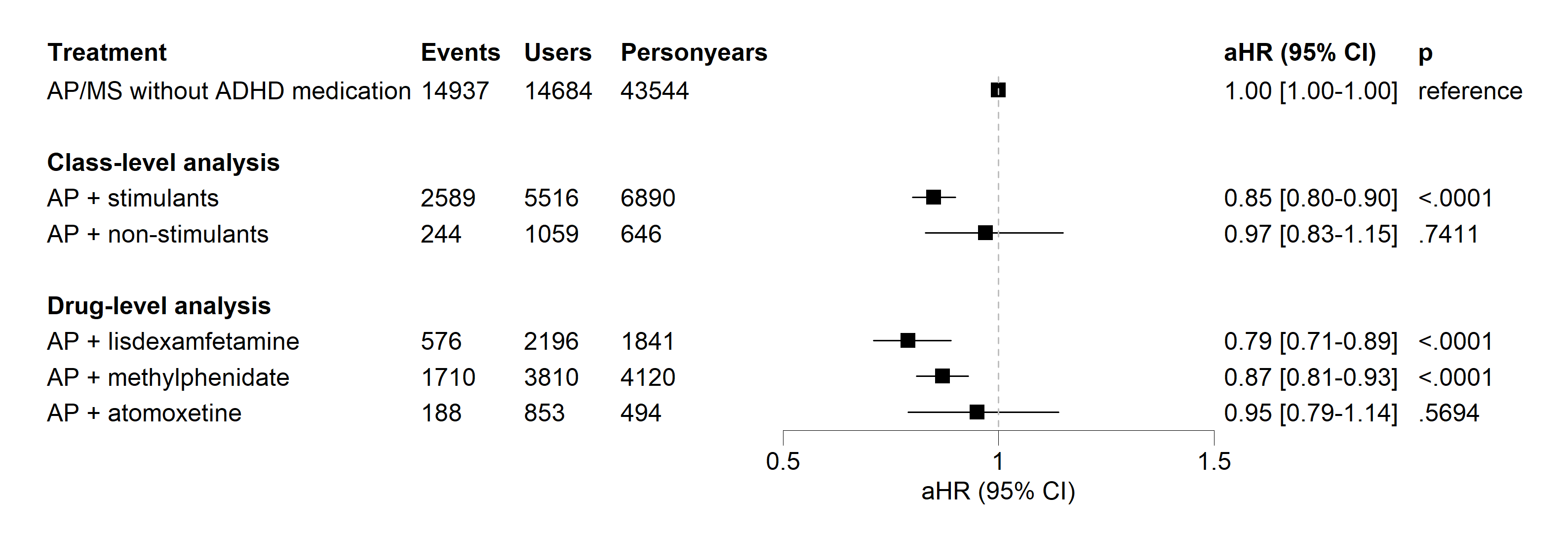
**

**Figure S3.** Sensitivity analysis for risk of psychiatric hospitalizations associated with use of attention-deficit hyperactivity disorder (ADHD) treatment in addition to treatment antipsychotic medications in within-individual analysis. The reference category was use of bipolar disorder treatment (i.e., antipsychotics or mood stabilizers [AP/MS]) without concomitant ADHD medications

*Abbreviations:* ADHD=attention-deficit/hyperactivity disorder, aHR=adjusted hazard ratio, AP=antipsychotic medications, AP/MS=antipsychotic and/or mood-stabilizer medication, CI=confidence interval, MS=mood-stabilizers.

**
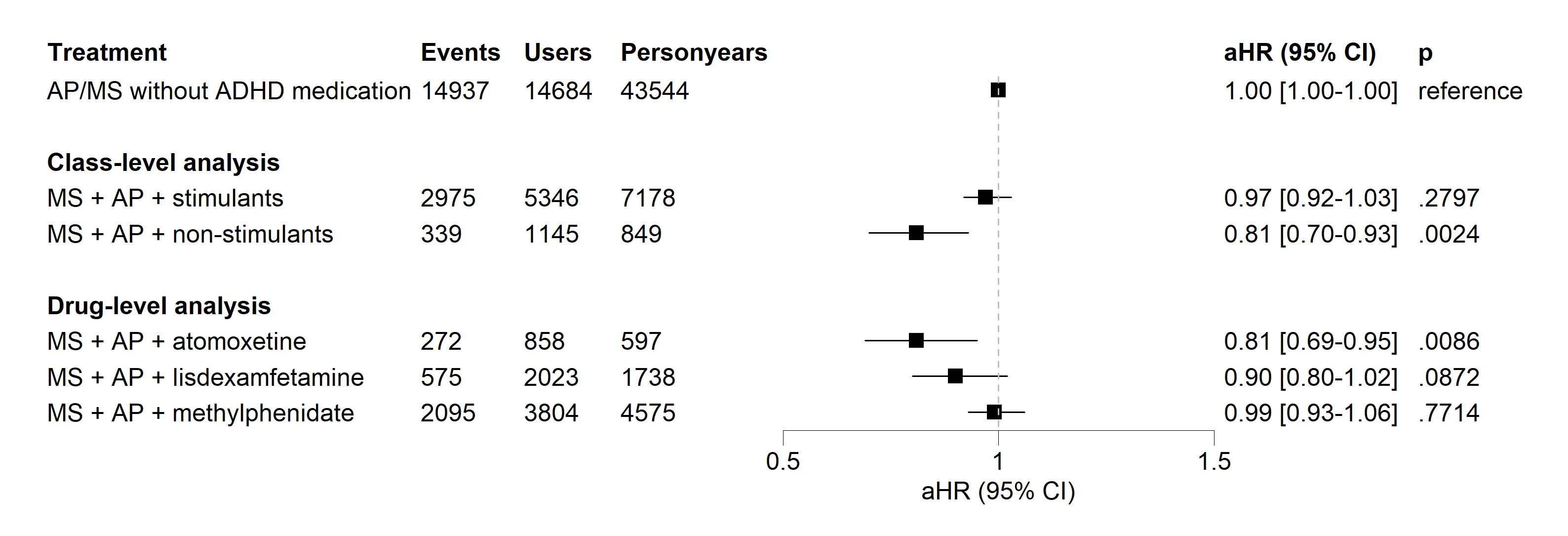
**

**Figure S4.** Sensitivity analysis for risk of psychiatric hospitalizations associated with use of attention-deficit hyperactivity disorder (ADHD) treatment in addition to treatment with mood-stabilizers and antipsychotics in within-individual analysis. The reference category was use of bipolar disorder treatment (i.e., antipsychotics or mood stabilizers [AP/MS]) without concomitant ADHD medications

*Abbreviations:* ADHD=attention-deficit/hyperactivity disorder, aHR=adjusted hazard ratio, AP=antipsychotic medications, AP/MS=antipsychotic and/or mood-stabilizer medication, CI=confidence interval, MS=mood-stabilizers.


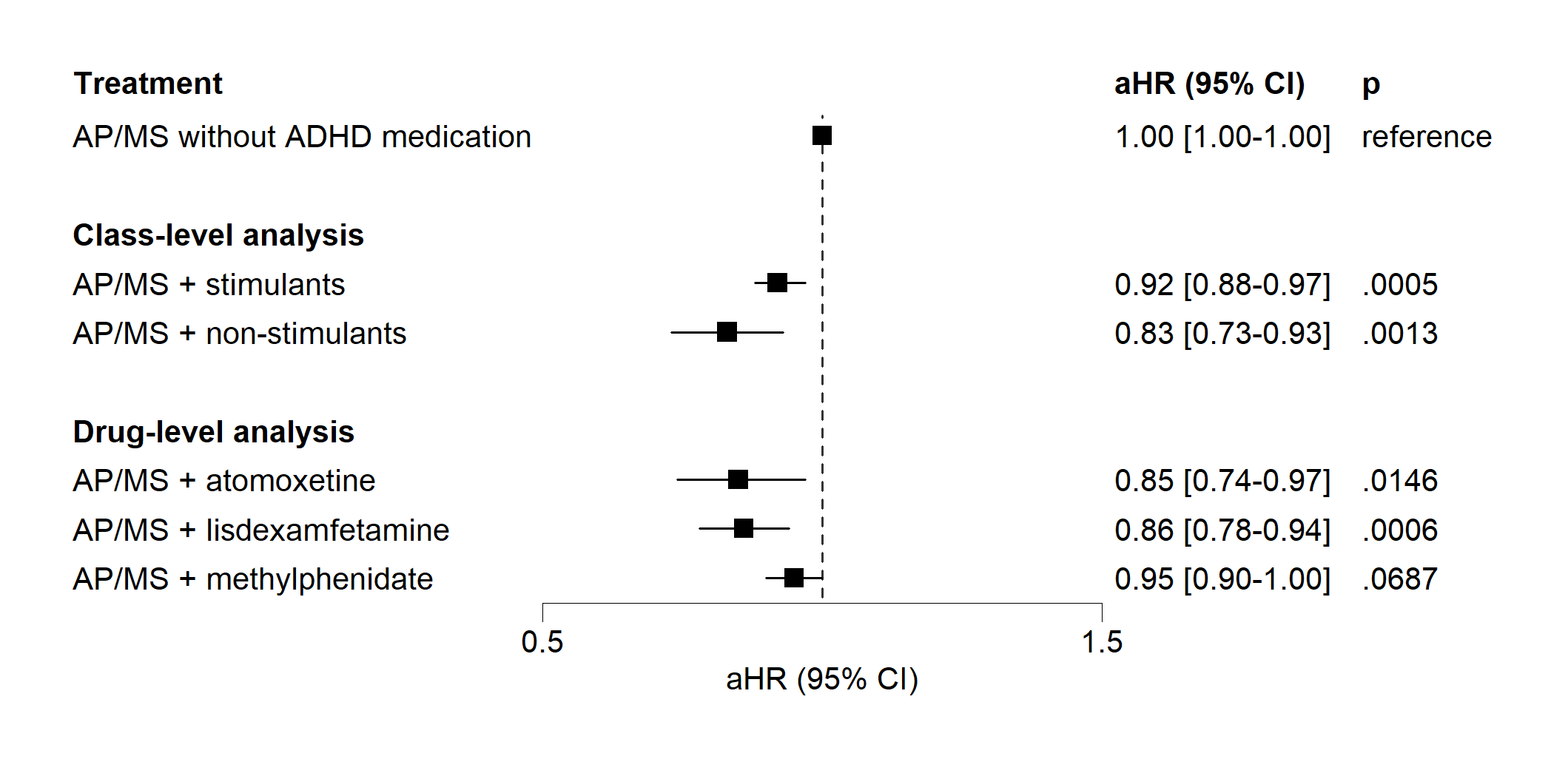


**Figure S5.** Sensitivity analysis for risk of psychiatric hospital admissions for treatment periods of ADHD medication in addition to antipsychotics or mood stabilizers (AP/MS) compared to treatment periods with AP/MS alone without any ADHD medication in within-individual analysis after omitting the first 30 days of exposure and non-exposure periods in the whole cohort

*Abbreviations:* ADHD=attention-deficit/hyperactivity disorder, aHR=adjusted hazard ratio, AP=antipsychotic medications, AP/MS=antipsychotic and/or mood-stabilizer medication, CI=confidence interval, MS=mood-stabilizers.

**
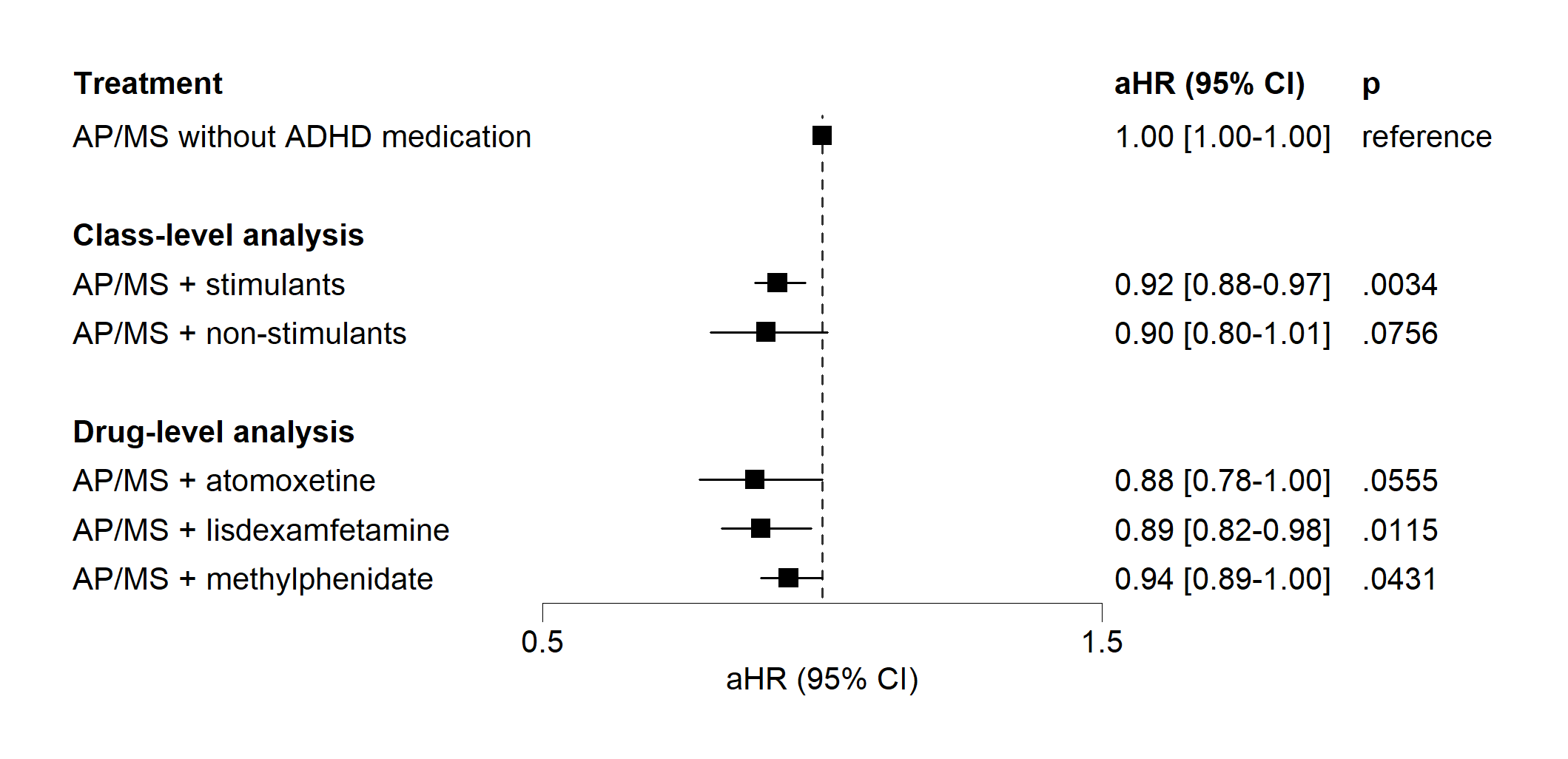
**

**Figure S6.** Sensitivity analysis for risk of psychiatric hospital admissions for treatment periods of ADHD medication in addition to antipsychotics or mood stabilizers (AP/MS) compared to treatment periods with AP/MS alone without any ADHD medication in within-individual analysis by restricting the analysis to time periods after the first observed ADHD medication use

*Abbreviations:* ADHD=attention-deficit/hyperactivity disorder, aHR=adjusted hazard ratio, AP=antipsychotic medications, AP/MS=antipsychotic and/or mood-stabilizer medication, CI=confidence interval, MS=mood-stabilizers.

‘


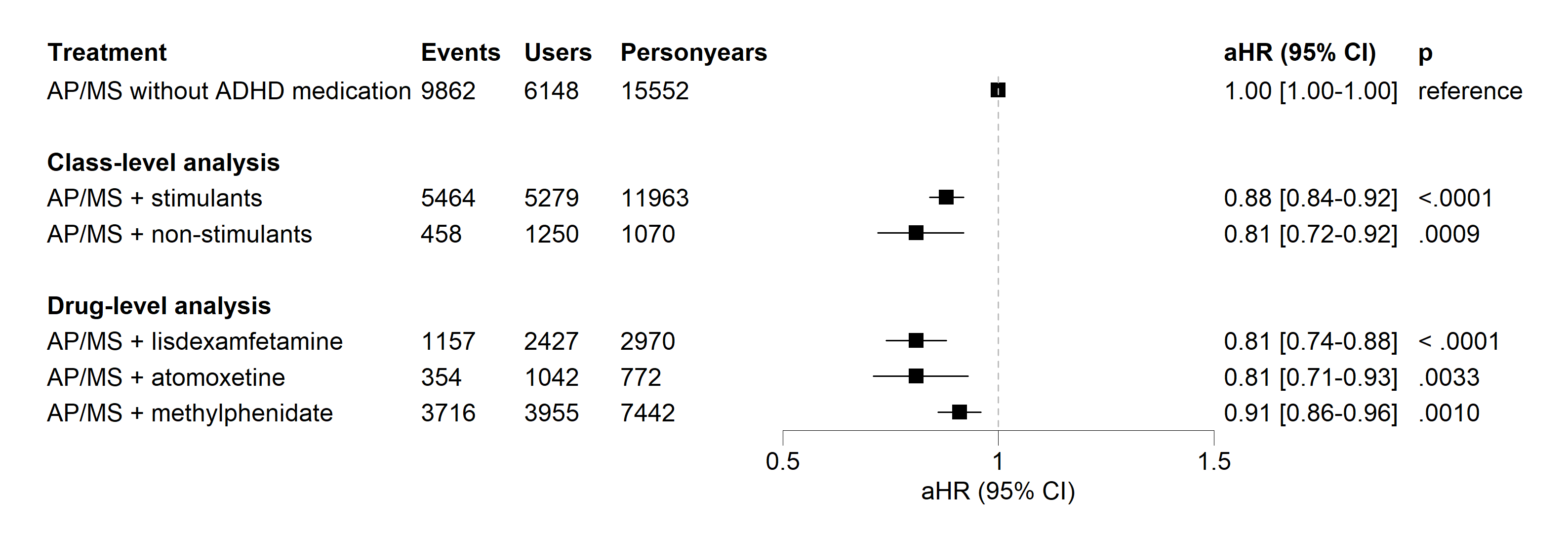


**Figure S7**. Sensitivity analysis for risk of psychiatric hospitalizations associated with use of add-on stimulant and non-stimulant medications in compared to treatment periods with antipsychotic and/or mood-stabilizer alone (AP/MS) on group-level analysis in within-individual models including individuals who had comorbid substance use disorder during the cohort

The subgroup was formed by individuals who had SUD. One might receive related ICD-code (F10-F19, except for F17) either before the original cohort entry (i.e., BD diagnosis) or during the follow-up. Therefore, the number of individuals is higher than the number of those at the original cohort entry. In this subgroup analysis, the follow-up started when an individual had both BD and SUD diagnoses: The 1st of January 2006 for those who had both BD and SUD before 2006, or the specific time point when both conditions were present.

Note. ADHD=attention-deficit/hyperactivity disorder, aHR=adjusted hazard ratio, AP=antipsychotic medication, AP/MS=antipsychotic and/or mood-stabilizer medication, BD=bipolar disorder, CI=confidence interval, ICD=International Classification of Diseases, MS=mood stabilizer, SUD=substance use disorder.


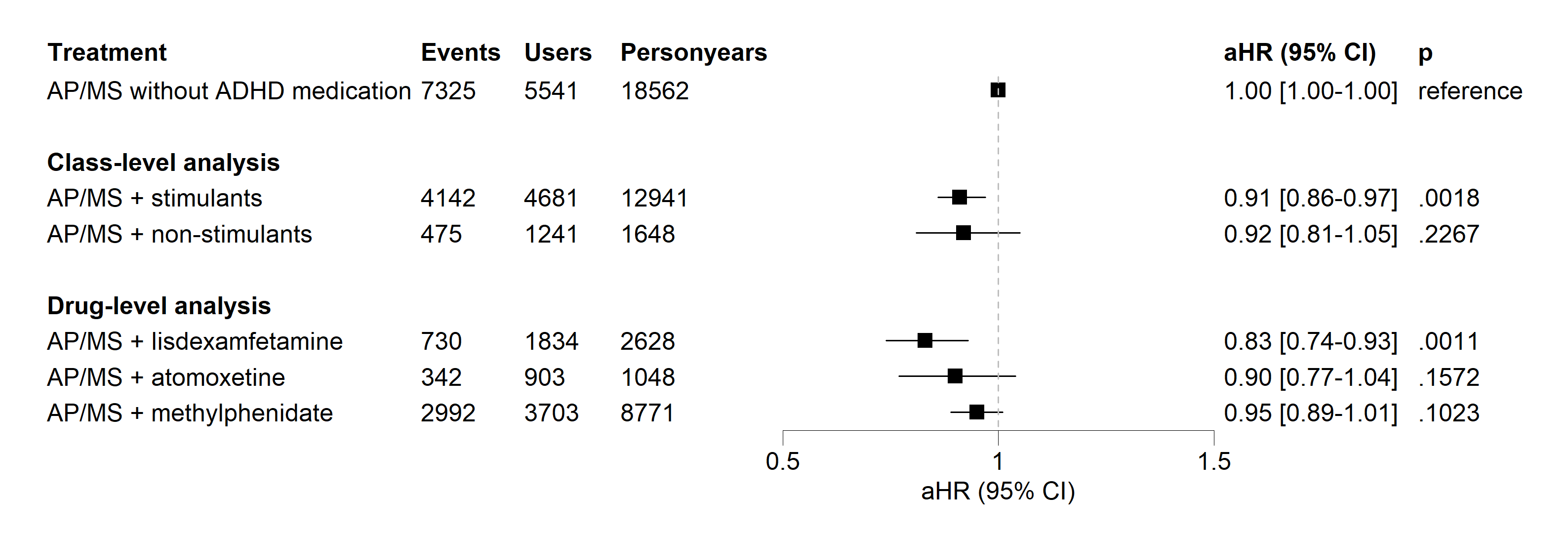


**Figure S8**. Sensitivity analysis for risk of psychiatric hospitalizations associated with use of add-on stimulant and non-stimulant medications compared to treatment periods with antipsychotic and/or mood-stabilizer alone (AP/MS) on group-level analysis in within-individual models including individuals who received disability pension during the cohort

The subgroup was formed by individuals who received disability pension. One might receive disability pension either before the original cohort entry (i.e. BD diagnosis) or during the follow-up. Therefore, the number of individuals is higher than the number of those at the original cohort entry. In this subgroup analysis, the follow-up started when one individual had both BD diagnosis and disability pension: The 1st of January 2006 for those who had both BD and disability pension before 2006, or the specific time point when both conditions were present.

*Abbreviations*: ADHD=attention-deficit/hyperactivity disorder, aHR=adjusted hazard ratio, AP=antipsychotic medication, AP/MS=antipsychotic and/or mood-stabilizer medication, BD=bipolar disorder, CI=confidence interval, MS=mood stabilizer.


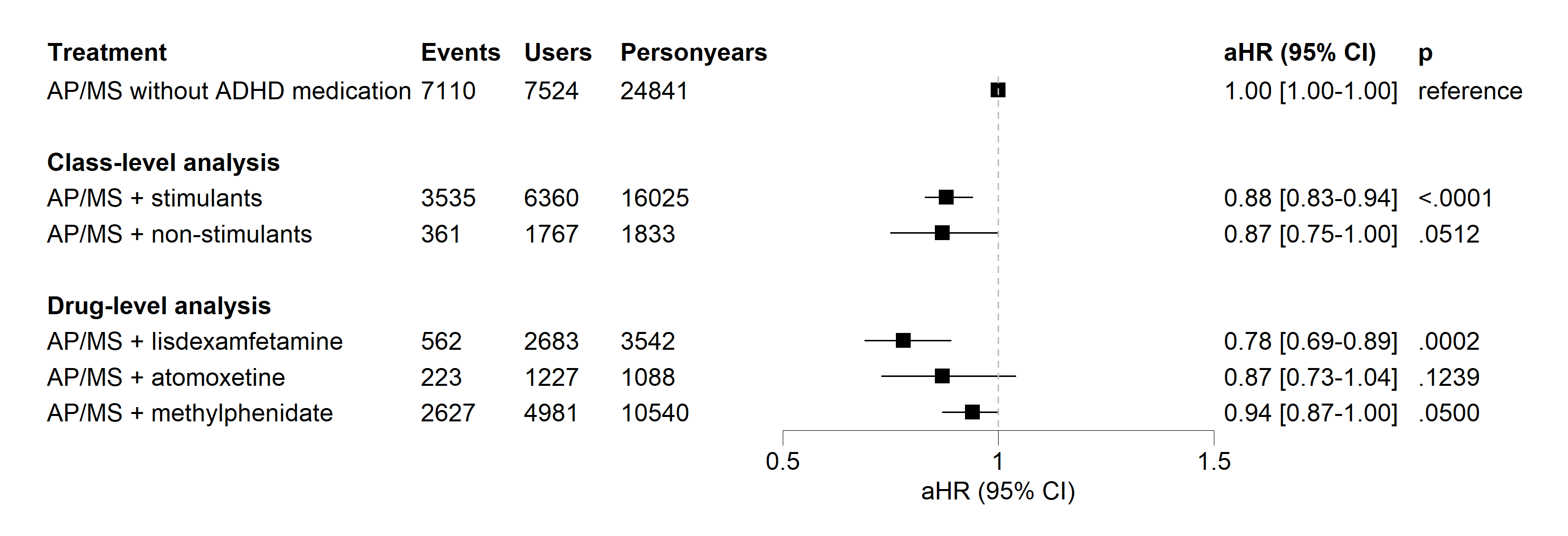


**Figure S9.** Risk of psychiatric hospital admissions for treatment periods of ADHD medication in addition to antipsychotics or mood stabilizers (AP/MS) compared to treatment periods with AP/MS alone without any ADHD medication in within-individual analysis including individuals aged ≥30 years at the cohort entry

*Note.* Within-individual analysis for treatment periods with both ADHD and BD treatments compared to treatment periods without any ADHD treatment: The number of users was not mutually exclusive for any treatment category since one individual might contribute to various treatment categories in different timeframes. The AP/MS category included all combinations or monotherapies of AP and MS medications pooled together. The reference category was treatment periods with BD treatment (i.e., AP and/or MS treatment) without ADHD medications.

*Abbreviations:* ADHD=attention-deficit/hyperactivity disorder, aHR=adjusted hazard ratio, AP=antipsychotic medication, AP/MS=antipsychotic and/or mood-stabilizer medication, BD=bipolar disorder, CI=confidence interval, MS=mood stabilizer.


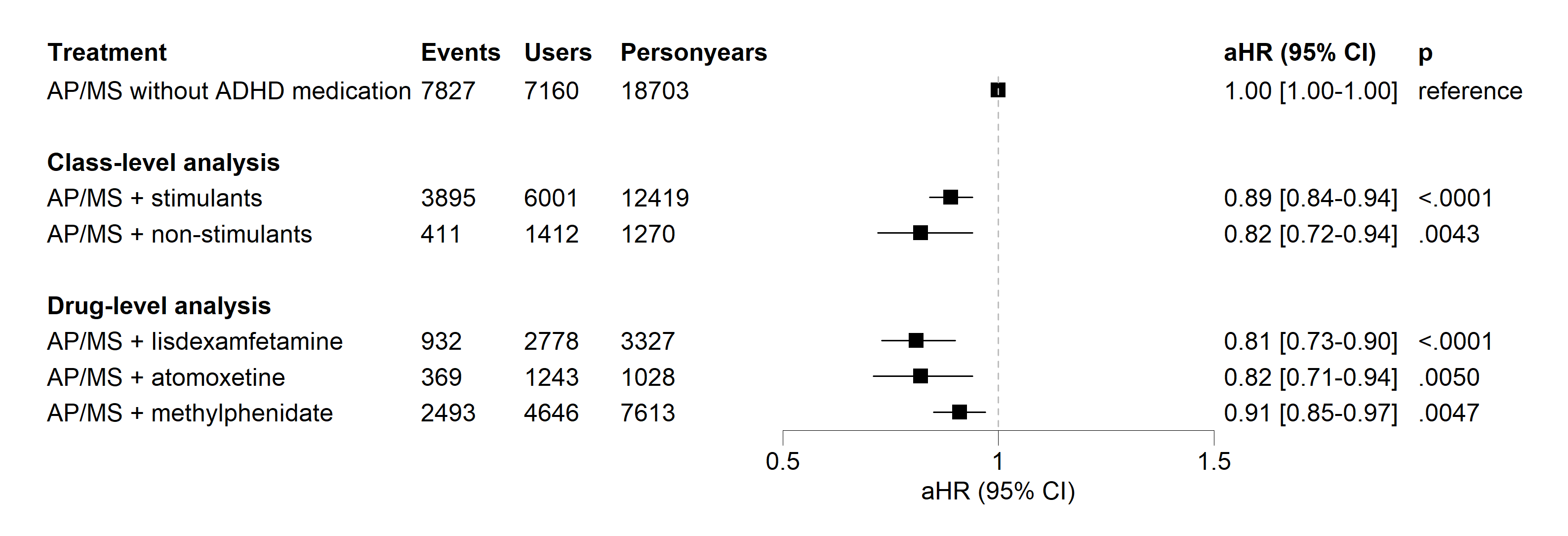


**Figure S10.** Risk of psychiatric hospital admissions for treatment periods of ADHD medication in addition to antipsychotics or mood stabilizers (AP/MS) compared to treatment periods with AP/MS alone without any ADHD medication in within-individual analysis including individuals aged 16-29 years at the cohort entry

*Note.* Within-individual analysis for treatment periods with both ADHD and BD treatments compared to treatment periods without any ADHD treatment: The number of users was not mutually exclusive for any treatment category since one individual might contribute to various treatment categories in different timeframes. The AP/MS category included all combinations or monotherapies of AP and MS medications pooled together. The reference category was treatment periods with BD treatment (i.e., AP and/or MS treatment) without ADHD medications.

*Abbreviations:* ADHD=attention-deficit/hyperactivity disorder, aHR=adjusted hazard ratio, AP=antipsychotic medication, AP/MS=antipsychotic and/or mood-stabilizer medication, BD=bipolar disorder, CI=confidence interval, MS=mood stabilizer.

**
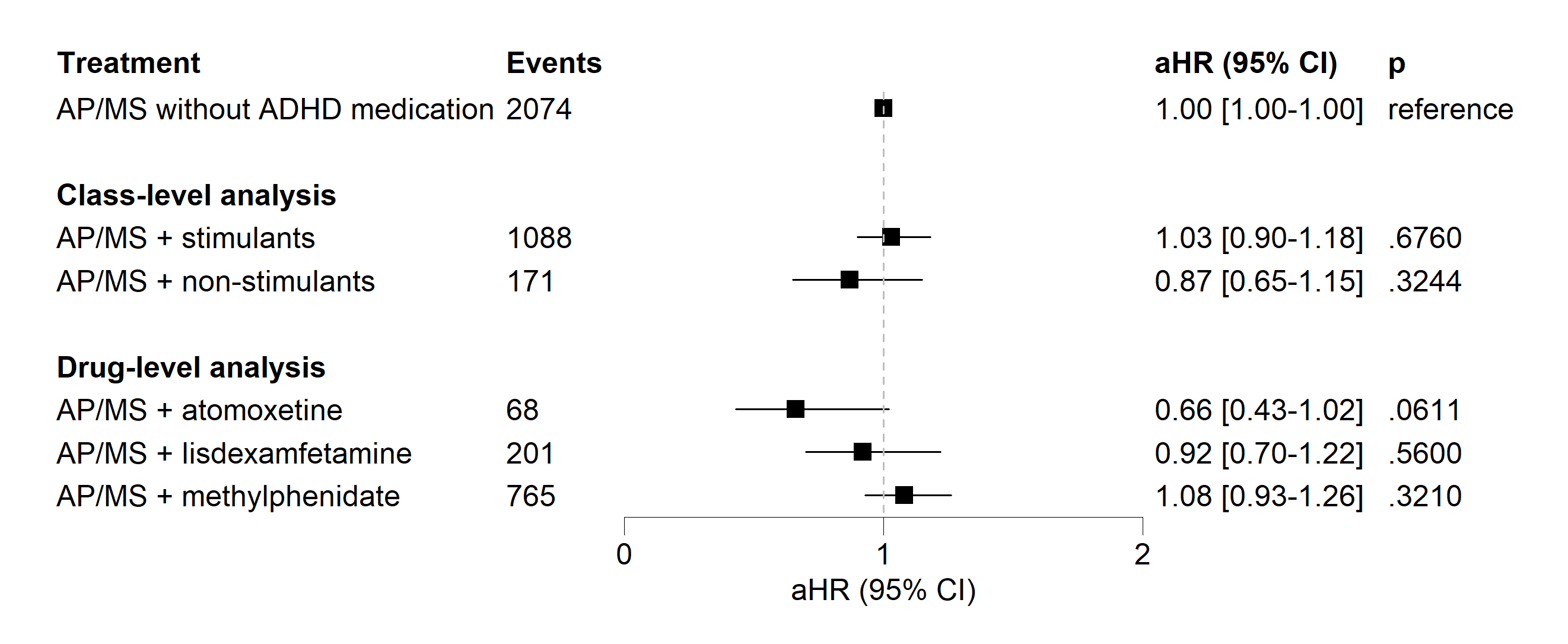
**

**Figure S11.** Risk of somatic hospital admissions for treatment periods of ADHD medication in addition to antipsychotics or mood stabilizers (AP/MS) compared to treatment periods with AP/MS alone without any ADHD medication in within-individual analysis including individuals aged ≥30 years at the cohort entry

*Note.* Within-individual analysis for treatment periods with both ADHD and BD treatments compared to treatment periods without any ADHD treatment: The number of users was not mutually exclusive for any treatment category since one individual might contribute to various treatment categories in different timeframes. The AP/MS category included all combinations or monotherapies of AP and MS medications pooled together. The reference category was treatment periods with BD treatment (i.e., AP and/or MS treatment) without ADHD medications.

*Abbreviations:* ADHD=attention-deficit/hyperactivity disorder, aHR=adjusted hazard ratio, AP=antipsychotic medication, AP/MS=antipsychotic and/or mood-stabilizer medication, BD=bipolar disorder, CI=confidence interval, MS=mood stabilizer.


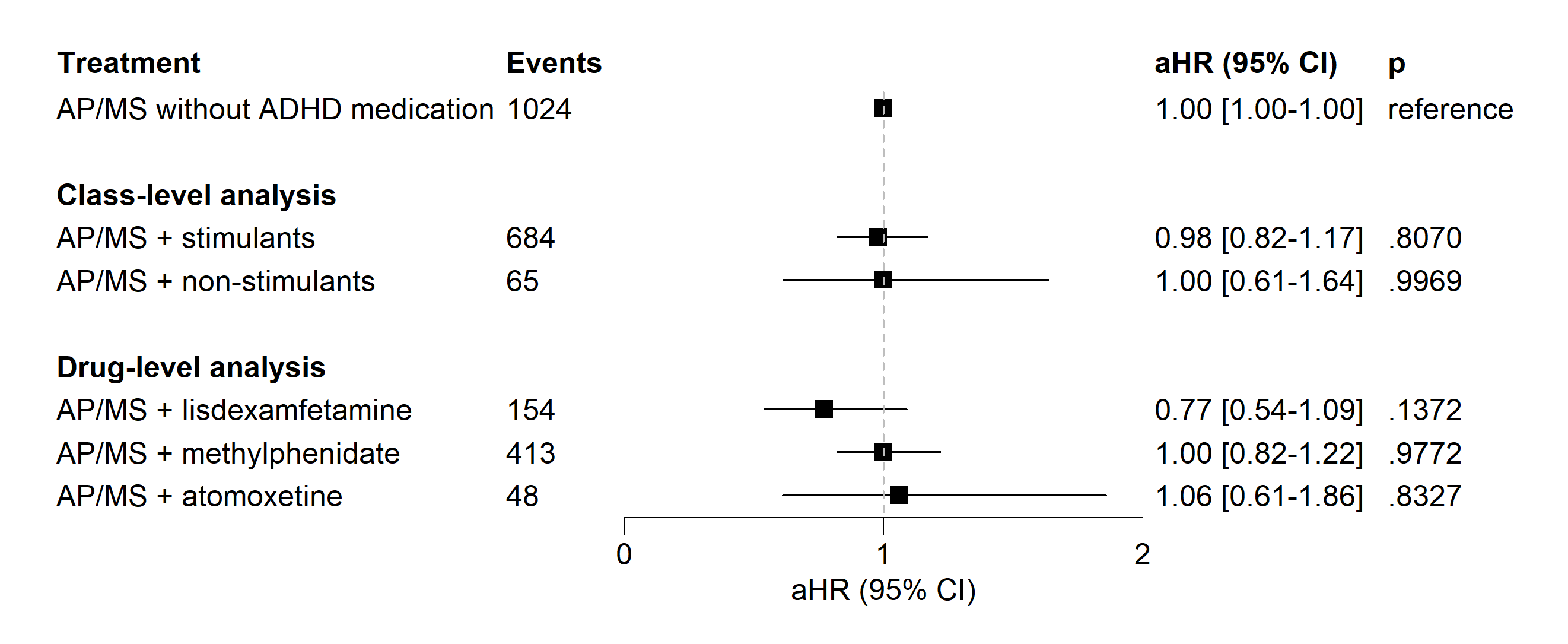


**Figure S12.** Risk of somatic hospital admissions for treatment periods of ADHD medication in addition to antipsychotics or mood stabilizers (AP/MS) compared to treatment periods with AP/MS alone without any ADHD medication in within-individual analysis including individuals aged 16-29 years at the cohort entry

*Note.* Within-individual analysis for treatment periods with both ADHD and BD treatments compared to treatment periods without any ADHD treatment: The number of users was not mutually exclusive for any treatment category since one individual might contribute to various treatment categories in different timeframes. The AP/MS category included all combinations or monotherapies of AP and MS medications pooled together. The reference category was treatment periods with BD treatment (i.e., AP and/or MS treatment) without ADHD medications.

*Abbreviations:* ADHD=attention-deficit/hyperactivity disorder, aHR=adjusted hazard ratio, AP=antipsychotic medication, AP/MS=antipsychotic and/or mood-stabilizer medication, BD=bipolar disorder, CI=confidence interval, MS=mood stabilizer.

**
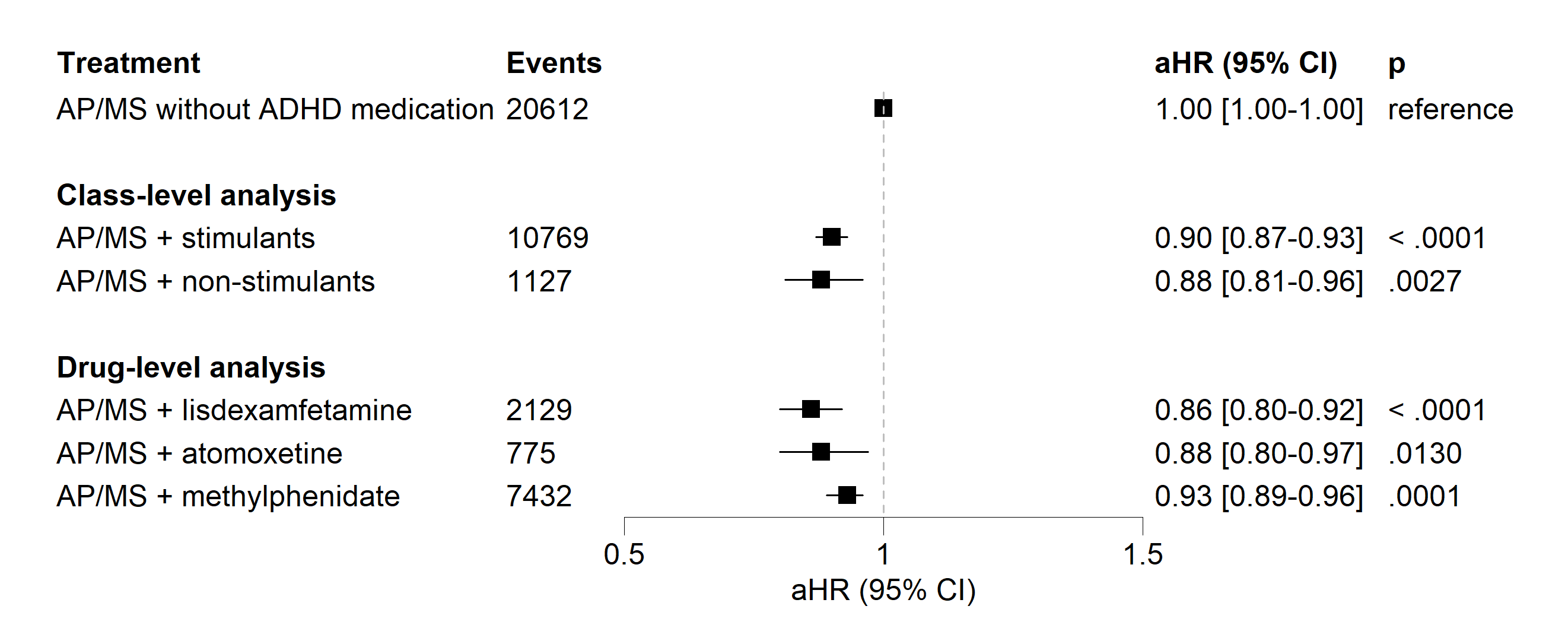
**

**Figure S13.** Risk of all-cause hospitalization/ mortality for treatment periods of ADHD medication adjunctive to antipsychotics or mood stabilizers (AP/MS) compared to treatment periods with AP/MS alone without any ADHD medication in within-individual analysis.

*Note.* Within-individual analysis for treatment periods with both ADHD and BD treatments compared to treatment periods without any ADHD treatment: The number of users was not mutually exclusive for any treatment category since one individual might contribute to various treatment categories in different timeframes. The AP/MS category included all combinations or monotherapies of AP and MS medications pooled together. The reference category was treatment periods with BD treatment (i.e., AP and/or MS treatment) without ADHD medications.

As there were 25 comparisons in the main analyses, the threshold for significance after family-wise error rate correction was set at p < 0.0020 (i.e. 0.05/25) level.

*Abbreviations:* ADHD=attention-deficit/hyperactivity disorder, aHR=adjusted hazard ratio, AP=antipsychotic medication, AP/MS=antipsychotic and/or mood-stabilizer medication, BD=bipolar disorder, CI=confidence interval, MS=mood stabilizer
